# New Heteroleptic 3D Metal Complexes: Synthesis, Antimicrobial and Solubilization Parameters

**DOI:** 10.3390/molecules25184252

**Published:** 2020-09-16

**Authors:** Muhammad Babar Taj, Muneera D. F. Alkahtani, Uzma Ali, Ahmad Raheel, Walla Alelwani, Afnan M. Alnajeebi, Nouf Abubakr Babteen, Sadia Noor, Heba Alshater

**Affiliations:** 1 Department of Chemistry, Islamia University of Bahawalpur, Bahawalpur 63100, Pakistan; 2 Department of Chemistry, University of Sahiwal, Sahiwal 57000, Pakistan; 3 Department of Biology, College of Science, Princess Nourah Bint Abdulrahman University, Riyadh 11675, Saudi Arabia; 4 Department of Chemistry, Quaid-e-Azam University, Islamabad 44000, Pakistan; uzmaali@hotmail.com (U.A.); ahmadraheel001@gmail.com (A.R.); 5 Department of Biochemistry, Faculty of Science, University of Jeddah, Jeddah 80203, Saudi Arabia; welwani@uj.edu.sa (W.A.); Amalnajeebi@uj.edu.sa (A.M.A.); Nababteen@uj.edu.sa (N.A.B.); 6 Department of Chemistry, University of Agriculture, Faisalabad 38040, Pakistan; 7 Department of Forensic Medicine and Clinical Toxicology, Menoufia University, Shbien El-Kom 32511, Egypt; hebaalshater@yahoo.com

**Keywords:** mixed chelation, electronic spectroscopy, stability constants, antimicrobial, micellar solubilization

## Abstract

The microbial resistance to current antibiotics is increasing day by day, which in turn accelerating the development of new effective drugs. Several studies have proved the high antimicrobial potential of the interaction of several organic ligands with a variety of metal ions. In the present study, a conventional method has been adopted in the synthesis of twelve new heteroleptic complexes of cobalt (II), nickel (II), copper (II) and zinc (II) using three aldimines, namely, (HL_1_ ((*E*)-2-((4-chloro-2-hydroxybenzylidene)amino)-3,4-dimethyl-5-phenylcyclopent-2-en-1-one), HL_2_ ((*Z*)-3-((4-chlorobenzylidene)amino)-4-hydroxy-5-nitrobenzenesulfonic acid) HL_3_ (2,2′-((1,2-phenylenebis(azaneylylidene))bis(methaneylylidene))diphenol)) as primary ligands, while phenyl glycine was the secondary ligand. The synthesized compounds were characterized by UV-vis, IR and multinuclear (^1^H and ^13^C) NMR spectroscopy, elemental analysis, and electrical conductance. The IR study revealed the coordination of the aldimine derivatives with the -OH and N atom of imine moiety. In contrary to this, the phenyl glycine coordinated to the metal ions via oxygen of carboxylate and nitrogen of the amino group. The spectroscopic analysis unveiled the tetrahedral geometry of the synthesized metal (II) complexes, except for ligand HL_3_ which exhibited octahedral geometry. The synthesized compounds generally showed antibacterial activity for all microbes, except Ni (II) complexes lacking sensitivity. Furthermore, to access the bioavailability, the synthesized complexes were screened for their solubilization in the micellar media of sodium lauryl sulphate. The metal complex–surfactant interaction was revealed by UV-vis spectroscopy and electrical conductivity measurements.

## 1. Introduction

Biomedical specialists aim to develop more potent and aggressive pharmacological alternatives due to the significant increase in drug-resistant microbial infections [1]. Researchers have embraced the potentials of natural products as effective pharmacological substitutes to combat microbial infections [2].

The role of transition metal complexes formation is considerably acknowledged in biological systems due to their ability to construct mix ligand complexes and their role in complicated equilibrium processes, especially enzyme–substrate interactions [3]. The chelates are formed in biological fluids through one or more coordination sites of ligands having different functional groups, and they play a significant role in detoxification and remediation of metal pollutants [4]. The complexation behavior and stability of many ligands with divalent transition metal ions have been widely investigated [5]. Copper is an essential component of numerous endogenous antioxidant enzymes as well as oxidative processes [6]. The role of cobalt in biochemical processes is extensively studied and is the component of many coenzymes and vitamin B_12_ [7]. Nickel is an essential metal that performs numerous roles in biochemistry of microorganisms, animals and plants [8]. Zinc is important for its catalytic role or exclusively structural role in maintaining the configuration of protein for most of its contribution [9]. Zinc ions can adapt to a variety of binding arrangements and different combinations of ligands, subsequently depicting a broad range of reactivity and functions [10].

In coordination chemistry, nitrogen-donor ligands, with strong redox stability and quite easy access to functionalization, have been extensively explored for the formation of heteroleptic complexes, that is, metal species which have more than one type of ligand [11,12]. The biological potentials including cytotoxicity, genotoxicity, antineoplastic, antitumor and bactericidal attributes have been reported for the heteroleptic chelates encompassing the bidentate ligands [2,13]. The chelation in metal complexes plays an important role in improving their lipophilicity in comparison to their precursor ligands [14]. The involvement of heteroatoms of ligands during chelation with metallic ions augmented the biological potentials of biologically active compounds [15]. The metal complexes with aldimine ligands are becoming key medicinal compounds having a broad range of biological activities [16]. It is well known that amino acids play an important role in the transportation of metal in the blood through the tissues. Many copper complexes with amino acids have been reported as anti-tumor and anti-inflammatory drugs. Pt (II) complexes with an L-alanyl-based ligand have been reported as promising antiproliferative drugs [17]. The antioxidant, antibacterial, antifungal and antitumor activities of metal complexes have been studied very extensively [18].

Several metal complexes show limited therapeutic efficiency due to the low solubility in aqueous media, and the surfactants play a vital role in enhancing solubility of drugs and other additives. 

Surface active agents possess a polar head and a non-polar tail end, which allow them to self-aggregate in the form of colloidal size particles called micelles. These micelles mimic the mechanism of interfacial adsorption of additives on their surface. The critical micellar concentration (CMC) is the concentration point where the aggregates of surfactants start to form, and it is affected by changes in concentration, temperature, solvents, electrolytic additives, nature of counter ion, pH etc.

The extreme biological importance of mixed ligand complexes, especially those containing amino acids as ligands, motivated us to synthesize new heteroleptic 3D metal complexes. To the best of our knowledge, this is the first study on the synthesis, antimicrobial activity and solubilization factors of 3D heteroleptic complexes of aldimine and glycine.

## 2. Results and Discussion

### 2.1. Synthesis of the Heteroleptic Complexes

**Scheme 1:**
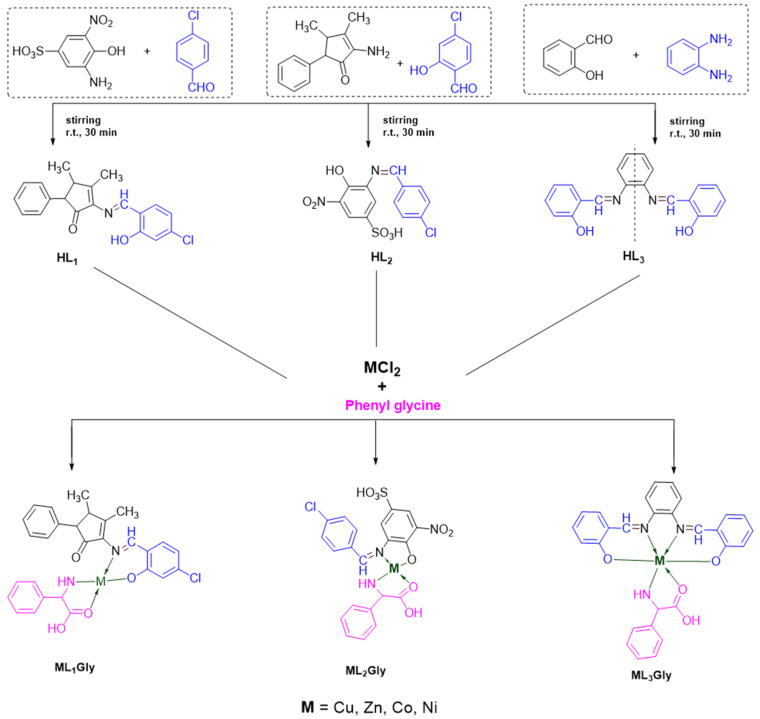
Synthesis of aldimine ligands (HL_1_–HL_3_) and metal complexes.

Three aldimine ligands were prepared by reacting separately three aromatic aldehydes with three different amines under reflux for 30 min, as reported in Scheme 1. From these reactions, three precipitates of the aldimines were obtained after cooling by filtration and used directly without further purification. The subsequent metal complexation was simply obtained by mixing the aldimine ligands (HL_1–3_) with equimolar amounts of phenyl glycine and the chlorides of Co (II), Ni (II), Cu (II) and Zn (II) (Scheme 1).

### 2.2. Characterization

All the synthesized heteroleptic complexes are colored amorphous, non-hygroscopic and air-stable solids which were soluble in DMF (dimethylformamide), DMSO (dimethyl sulfoxide) and partially soluble in methanol, ethanol, and chloroform. All the synthesized complexes were characterized based on the determination of melting points, molar conductance and spectrochemical analysis. The electrical molar conductance of the synthesized complexes was measured in DMSO (10^−3^ M) at ambient temperature. The low or high values of molar conductance obtained for the synthesized heteroleptic complexes of divalent metal ions Co (II), Ni (II), Cu (II) and Zn (II) indicated their nonelectrolyte or electrolytic behavior [19] (Scheme 1). The selected NMR and FTIR spectra of ligands and complexes are reported in Figures S1–S16.

### 2.3. Infrared Spectroscopic Analysis

The FTIR spectra (4000–400 cm^−1^) were recorded for the free aldimines, α-phenyl glycine, and their corresponding metal complexes. The FTIR spectrum of free phenyl glycine ligand has prominent peaks at 2975, 3167 for υ (-NH_2_) sym./ asym., 1355 for υ (COO^−^) asym., and 1576 for υ (COO^−^) sym. When the amino acid chelated the metal, significant changes were observed in υ (NH_2_) and υ (COO^−^) IR regions. It is important to point out here that free amino acids occur as zwitterions, and the IR spectra of these cannot be matched entirely with those of metal complexes, as amino acids in metal complexes do not occur as zwitterions. The amino acid showed symmetric and asymmetric vibrations due to υ (COO^−^), which shifted and changed in intensities in the complexes. Similarly, the symmetric and asymmetric band in -NH_2_ group also migrated to higher frequencies after chelating the metal ion with aldimine and the α-phenyl glycine ligand. Further evidence regarding the involvement of both the COO^−^ and NH_2_ groups in the complexes was assessed by the appearance of some weak IR bands in the far region at 500–600 cm^−1^ [20] due to υ (M-N) and 600–700 cm^−1^ [21] due to υ (M-O), respectively, being absent in the free ligands.

### 2.4. IR Analysis of HL_1_

The characteristic band for carbonyl group (C=O) appeared at 1647 cm^−1^ and for aldimine group (–CH=N) appeared at 1591 cm^−1^ in the spectrum of the free aldimine ligand, which was found at lower frequencies after metal complexation (~10–30 cm^−1^). This shift indicated the involvement of the nitrogen atom of the aldimine group in the coordination. The O-H stretching band of –COOH was absent in the IR spectra of all metal complexes after deprotonation. Some new bands with weak intensities at 646–614 (M-O) and 597–459 cm^−1^ (M-N) were also seen in the complexes. The less covalent character in the M-O bond relative to M-N bond caused the appearance of metal–oxygen stretching bands to bond at high-frequency regions.

### 2.5. IR Analysis of HL_2_

In the case of ligand 2, the -OH stretching vibration was spotted at 3379 cm^−1^ which vanished after complex formation, indicating its contribution towards M-O bond formation. The aldimine characteristic –C=N stretching vibration of the free ligand was witnessed at 1625 cm^−1^ which shifted to 1651, 1595 and 1622 cm^−1^ in complexes, indicating the involvement M-O co-ordinate covalent bond. The symmetric and asymmetric vibrations due to –COO^−^ were observed at 1294–1377 and 1562–1585 cm^−1^ correspondingly. The bands at 597–644 and 441–486 cm^−1^ were designated to the characteristic M-O and M-N bonds in the complexes. 

### 2.6. IR Analysis of HL_3_

The IR analysis of ligand HL_3_ demonstrated the characteristic band due to the aldimine functional group (–CH=N–) at 1609 cm^−1^, which then shifted to higher wavenumbers in complexes. The υ (O-H) band of free ligand was present at 3453 cm^−1^, and it disappeared in the spectra of complexes. The characteristic bands due to secondary ligand (DL)-α-phenyl glycine were also observed. The asymmetric vibration bands due to carboxylic group of the amino acid at 1576 cm^−1^ in the free ligand were shifted to a lower frequency (~50 cm^−1^) in the spectra of the complexes. Similarly, the symmetric and asymmetric bands of the υ (–NH_2_) were observed at 3167–2975 cm^−1^. The evidence regarding contributions of aldimine and carboxylate after complexation was validated by weak bands at 500–600 and 600–700 cm^−1^ due to M-N and M-O bonds of complexes. 

### 2.7. Electronic Spectral Studies

The electronic spectra of synthesized complexes and ligands (10^−3^ M) were recorded after dissolving them in DMSO, and the results are presented in Table 1.

The free ligand HL_1_ exhibited strong absorption at 347 nm due to the n→π* transition of imine moiety. On complexation, the band shifted to higher wavelength regions and possibly due to the coordination of nitrogen with metals.

The free ligand HL_2_ confirmed the presence of a prominent and intense band at 358 nm and the low-intensity band at 441 nm due to the n→π* transition of aldimine and the lone pair electrons of its nitrogen atom. In cobalt complexes, two bands of same intensity were observed at 456 nm and 476 nm, and another at 690 nm due to ^4^A_2_(F)→^4^T_1_(P), ^4^A_2_(F)→^4^T_1_(F) and ^4^A_2_(F)→^4^T_2_(F) and transitions for d^7^ tetrahedral geometry of the complex. The nickel complex shows three bands at 675 nm, 507 nm, 406 nm assigned to ^3^T_1_g(F)→^3^T_2_g(F), ^3^T_1_g(F)→^3^A_2g_(F) and ^3^T_1g_(F)→^3^T_1g_(P) for Ni^2+^ d^8^ tetrahedral geometry of the complex. Similarly, in the copper complex, three bands, one of which at 680 nm was due to ^2^T_2g_→^2^E_g_ transition of Cu^2+^ d^9^ tetrahedral geometry of the complex, were generally found in square planar geometry when tetracoordinated, while other bands were due to the ligand-centered n→π* and charge transfer transitions. The zinc complex did not show such a d-d transition due to its complete d^10^ configuration, while a band 445 nm was observed due to the n→π* transition of aldimine moiety.

Ligand HL_3_ showed two major peaks due to n→ π*, π→π* of aldimine functionality and benzene π-bonding electrons. The spectra of complexes indicated some prominent peaks for charge transfer as well as d-d transitions. The brown-colored cobalt complex showed three bands at 332 nm, 419 nm and 732 nm assigned to ^4^T_1g_(F)→^4^T_2g_(F), ^4^T_1g_(F)→^4^A_2g_(F) and ^4^T_1g_(F)→^4^T_1g_(P) transitions, suggesting d^7^ octahedral geometry of the complex. The maroon-colored nickel complex showed three bands at 377 nm 475 nm and 640 nm assigned to ^3^A_2g_(F)→3T_1g_(P), ^3^A_2g_(F)→^3^T_1g_(F) and ^3^A_2g_→^3^T_2g_(F) transitions, suggesting d^8^ octahedral geometry of the complex. The blue-colored copper complex showed charge transfer bands and one d-d band at 657 nm assigned to ^2^E_g_→^2^T_2g_ transition due to d^9^ octahedral geometry of the complex with the ^2^D ground atomic term. In the case of zinc complexes, filled d^10^ configuration caused no d-d transitions; however, two peaks at 293 nm and 402 nm were spotted indicating intra-ligand and metal-to-ligand charge transfer. The selected electronic spectra of ligands and complexes are given in Figure S17.

### 2.8. Stability Constants of Heteroleptic Complexes

The spectrophotometric method was carried out for the determination of the stability constants of metal complexes of aldimine ligands. There was a 1:2 ratio (metal:ligand) in all complexes. To estimate the stability constant, the degree of dissociation of complex (α) was obtained from Job’s plot for the given ligands and the metal ion in solutions. By substituting the value of (α) in the equation mentioned below, the value of the stability constant (K_st_) was obtained.

K_st_ = (1−α)/m^m^ × n^n^ × (α)^m + n^(C)^m + n − 1^ (1)

The calculations were performed for the heteroleptic complexes of Co (II), Ni (II), Cu (II) and Zn (II) with the three aldimine ligands (HL_1_, HL_2_, HL_3_). The zinc complex of ligand HL_3_ had the lowest K_st_ value among all the synthesized complexes. Exceptionally, Cu (II) complexes of all three ligands appeared to have lower K_st_ values as compared to the other ones, although the stability of the complexes increased as the size decreased. It is well known that a complex with a stability constant with a smaller value is less stable than those with a higher value.

The results followed Irving and Williams’s order of stability constants for transition metal ions and are summarized in Table 2.

### 2.9. Applied Studies on Synthesized Complexes

#### 2.9.1. Antibacterial Activity

**Figure 1:**
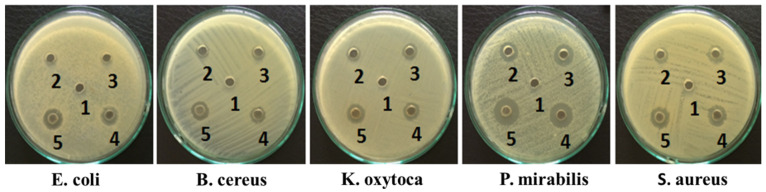
Antibacterial activity of CoL_1_Gly against different bacterial strains.

The results of the antibacterial analysis of the ligands HL_1–3_, glycine and their corresponding heteroleptic complexes with Co, Ni, Cu and Zn are summarized in Table 3. The synthesized compounds generally showed antibacterial activity for all microbes, except Ni (II) complexes lacking sensitivity against *Klebsiella oxytoca* and *Pseudomonas aureginosa*. As expected, the complexes were significantly more sensitive than glycine and their corresponding ligands HL_1–3_. All compounds exhibited exceptional potential against *E. coli* and *P. mirabilis*, with inhibition zones in the ranges of 13.2–29.7 and 16.0–30.0 mm. Furthermore, NiL_1_Gly, CoL_3_Gly, NiL_3_Gly demonstrated much-enhanced susceptibility as compared to ligands and glycine against *B. cereus*. These improved antibacterial activities of complexes may be attributed to the involvement of hyper-conjugation and induction of greater lipo solubility [22]. The polarity of metal ions diminished and caused delocalization of π-electrons, which, in turn, favored the penetration of complexes through the bacterial membrane, subsequently enhancing their activity [23,24]. In addition to this, cellular enzymes were neutralized by chelation, which plays a crucial role during metabolic activities of bacteria [24]. The cobalt complexes, particularly CoL_1_Gly (Figure 1), among all other counterparts, exhibited significant potential as antibacterial agents in comparison to the standard drug, making them suitable candidates for antibiotic drug research [25,26].

#### 2.9.2. Antifungal Activity

The outcomes of antifungal activities of the ligands HL_1–3_, glycine and their corresponding heteroleptic metal complexes are summarized in Table 4. The activity against *A. niger*, *A. flevus*, and *R. stolonifer* specified good to moderate inhibitory growth zones. The ligands inhibited the fungal strains and resulted in inhibitory zones of 14.3–29.4 mm in diameter. However, CoL_1–3_Gly complexes exhibited comparable activities to precursor ligands and other complexes against all three fungal strains, and cobalt ions were adsorbed to a greater extent onto the surface of fungal cell walls.

### 2.10. Interaction of Metal Complexes with Anionic Surfactant

The interaction of metal complexes with anionic surfactant was assessed by electrical conductivity and UV-visible spectroscopic analysis. In the first step, stock solutions of SLS (sodium lauryl sulfate) was prepared in water, and, then, conductivity measurements were performed with variable concentrations of surfactant at 298 K. These conductivity data were then plotted against SLS concentrations, and the value of CMC was determined from the point of inflection (Figure 1). The accurate value of CMC was assured by plotting the differential conductance against differential SLS concentration, and a reverse curve gave a more accurate value of CMC. There was a significant change in conductance in the pre-micellar region due to the presence of free anions and cations of surfactant but not in the post-micellar region, possibly due to micelle formation. CMC values for SLS were enhanced in all cases.

**Figure 2:**
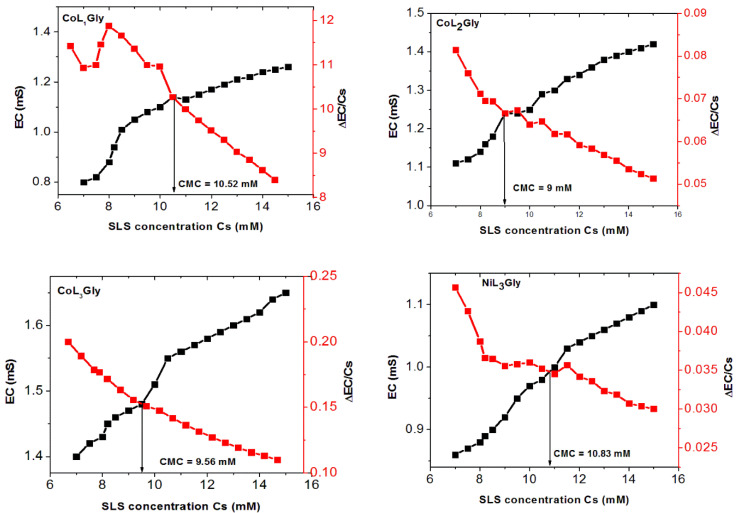
Plots of conductivity and differential conductivity vs. (sodium lauryl sulfate) SLS concentrations.

The values of CMC determined by conductivity data were crosschecked with UV-visible measurements. Initially, the λ_max_ of each complex was determined, and an absorbance was plotted against the concentration surfactant, as represented in Figure 2. Both techniques showed close agreement in terms of CMC values.

### 2.11. Electrical Conductivity Measurements

The micellar parameters of selected complexes and their CMC values were explored from simple and differential conductivity data. On gradual dilution, a noticeable variation in conductivity was observed. The value of critical micelle concentration (CMC) was determined from plots of electrical conductivity (EC) against SLS concentrations (CS) (Figure 2) [27,28].

A significant interaction was observed in the complexes of cobalt with all ligands and nickel with HL_3_ in the micellar media of SLS, and their micellar parameters are presented in Table 5. The negative values of ∆G_m_ indicated exothermicity of micellization as well as stability and spontaneity of this system. CoL_3_Gly/SLS was the most stable system, and its micellization was spontaneous as compared to the other complexes.

### 2.12. UV-Visible Spectroscopic Measurements

**Figure 3:**
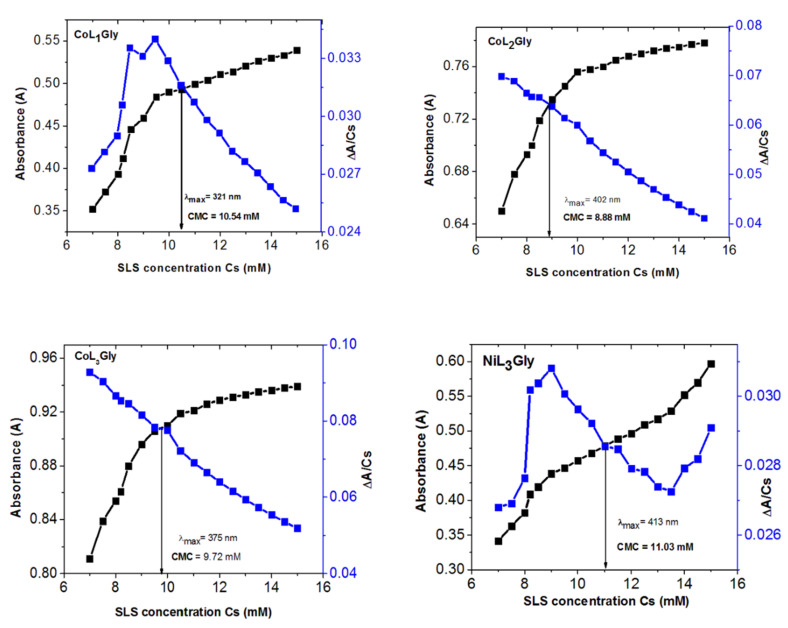
Plots of simple and differential absorbance vs. SLS concentrations.

Interaction of complexes with SLS micelles was assessed from simple and differential UV-visible spectroscopic data. The maximum absorbance (λ_max_) of the complexes in the absence and presence of various SLS concentrations was measured, and the extent of interaction was determined, as presented in Figure 3, whereas the partitioning and binding parameters are reported in Table 6.

The location of metal complexes in the surfactant depended on the hydrophobic and electrostatic interactions, and the results confirmed the partitioning in the proximal regions of the micelles. The molecules of metal complexes interacted with micelles and absorbance increase with increasing concentration of the surfactant. A bathochromic shift in the maximum absorbance was mainly due to significant interactions between complexes and surfactant molecules [27,28,29,30].

**Figure 4:**
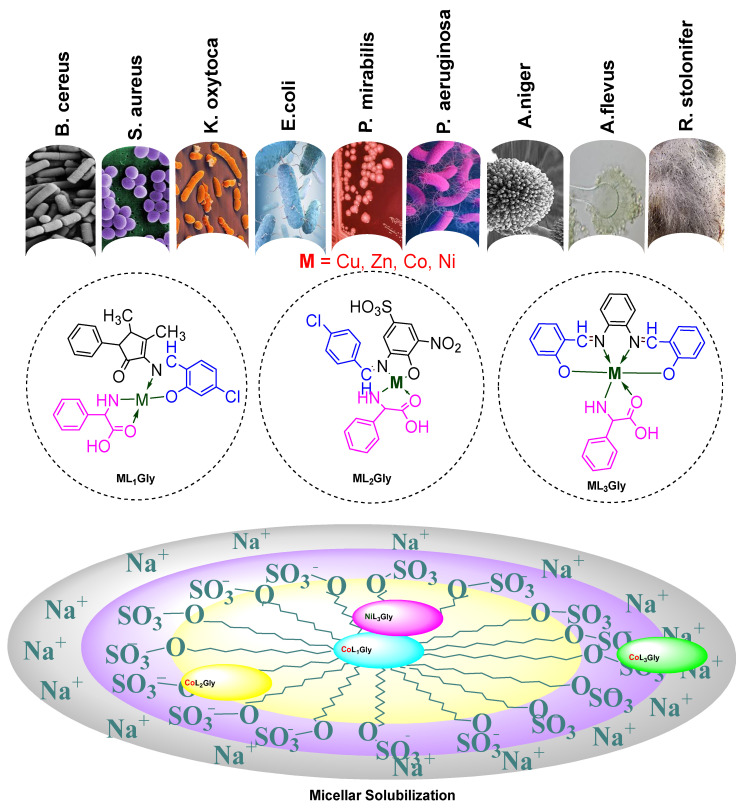
Proposed locus of metal complexes in the micelles of SLS.

The SLS structure breaking effect caused an increment in the CMC of surfactant in the presence of the complexes [27]. The penetration of complex molecules into the micelles increased the values of CMC in comparison to that of pure SLS. The involvement of intermolecular hydrogen bonding between water and metal complexes caused adsorption in the periphery of micelles [27]. The molecules of CoL_3_Gly resided near the outer region of the micelles, and those of CoL_1_Gly were positioned near the inner core region of micelles, as depicted in Figure 4.

## 3. Materials and Methods 

### 3.1. Chemicals and Instruments

All analytical grade chemicals were purchased from Sigma Aldrich and used as such during this investigation, and all the solvents were used after distillation and purification. Spectrophotometric measurements were taken on a Shimadzu UV-1700 Double Beam Scanning UV-Vis Spectrophotometer with 1.00 cm cell and a pH-meter (Sartorius Professional Meter PP-15, Duisburg Germany).

Infrared spectra were recorded on Perkin Elmer FTIR spectrophotometer (Perkin-Elmer, USA). The ^1^H-NMR (300 MHz) and ^13^C-NMR (75.43 MHz) spectra were recorded on Bruker AM-250 spectrometer (Bruker, Billerica, MA, USA) in CDCl_3_ and DMSO solution using TMS as the internal standard. Elemental analysis was performed by PerkinElmer 7300 DV and Leco (PerkinElmer, Inc., Shelton, CT, USA); CHN-932 elemental analyzer (Leco CHNS 932, Calgary, Canada) and the electronic sensitive balance (Shimadzu, Kyoto, Japan) were used during the study to find the stoichiometry and formation constant.

### 3.2. Synthesis of Aldimine Ligands (HL_1–3_)

Three aldimine ligands were prepared and subsequently used to synthesize complexes with Co (II), Ni (II), Cu (II) and Zn (II). The detailed physico-chemical analysis is given in Tables S1–S3.

#### 3.2.1. (*E*)-2-((4-chloro-2-hydroxybenzylidene)amino)-3,4-dimethyl-5-phenylcyclopent-2-en-1-one (HL_1_)

This ligand was prepared by mixing the ethanolic solution (25 mL) of 4-amino-2,3-dimethyl-1-phenyl-3-pyrozoline-5-one (2.03 g, 10 mmol) with an ethanolic solution of (25 mL) 4-chloro-2-hydroxy benzaldehyde (1.56 g, 10 mmol) in methanol (5 mL). The mixture was placed in a 100 mL round bottom flask connected with an external condenser and refluxed for 30 min. Upon cooling on ice, yellow precipitates were formed, filtered, washed with ethanol, and stored in vacuo over silica gel.

Yellow amorphous solid, yield, 75%; m.p. 250–254 °C. Elemental analysis of CHN for C_20_H_18_ClNO_2_: C 70.69; H 5.34; N 4.12%. Found: C 70.65; H 5.28; N 3.90%. ^1^H-NMR spectrum (300 MHz, DMSO-*d*_6_) δ_H_, ppm: 2.46 (1H, s, Ph), 3.196 (s, Me), 3.35 (s, Me), 7.5 (3H, t, Ph *J* = 8.1 Hz), 7.8 (2H, d, Ph *J* = 8.7 Hz), 9.56 (1H, s, –CH=N–); ^13^C-NMR spectrum (75 MHz, DMSO-*d*_6_) δ_C_, ppm: 14.3 (C_20_, Me), 15.9 (C_19_, Me), 58.1 (C_10_), 126.1 (C_14_, Ph), 128 (C_12_, C_16_, Ph), 132, 129 (C_4_, C_6_, C_13_, C_15_, Ph), 130.6 (C_7_, C_3_, Ph), 131.9 (C_2_, Ph), 132.9 (C_8_), 136.6 (C_5_, Ph), 139.3 (C_11_, Ph), 146.8 (C_18_), 163.7 (C_1_ C=N), 187.6 (C^9^, C=O). Electrical Conductance (μS/cm) 3.55. 

#### 3.2.2. (*Z*)-3-((4-chlorobenzylidene)amino)-4-hydroxy-5-nitrobenzenesulfonic acid (HL_2_)

For preparing ligand HL_2_, the ethanolic solution (25 mL) 5-nitro-2-aminophenol-4-sulfonic acid (2.34 g, 10 mmol) was mixed with an ethanolic solution of (25 mL) of 4-chlorobenzaldehyde (1.4 g, 10 mmol) in methanol (5 mL). The mixture was placed in a 100 mL round bottom flask connected with an external condenser and refluxed for 30 min. Upon cooling on ice, brown precipitates were formed, filtered, washed with ethanol, and stored in vacuo over silica gel.

Brown amorphous solid, yield, 79%; m.p. 125–128 °C. Elemental analysis of CHN for C_13_H_9_ClN_2_O_6_S: C 43.77; H 2.54; N 7.85%. Found: C 43.55; H 2.50; N 7.67%. ^1^H-NMR spectrum (300 MHz, DMSO-*d*_6_) δ_H_, ppm: 3.51 (1H, s, SO_3_H), 6.9 (1H, d, *J* = 8.7 Hz Ph), 7.2 (1H, t, *J* = 4.2 Hz, Ph), 7.4 (2H, d, *J* = 8.4 Hz, Ph), 7.8 (2H, d, *J* = 8.4 Hz, Ph), 8.5 (1H, s, C=N), 10 (1H, s, OH); ^13^C-NMR spectrum (75 MHz, DMSO-*d*_6_) δ_C_, ppm: 116.19 (C_12_), 116.23 (C_13_), 125.08 (C_4_, C_6_), 128.81 (C_3_, C_7_), 129.31 (C_2_), 130.11 (C-Cl, C_5_), 133.86 (C-NO_2_, C_10_), 135.82 (C_12_, C-SO_3_H), 138.22 (C_8_), 150.96 (C_9_, C-OH, Ph), 156.75 (C=N). Electrical Conductance (μS/cm) 2.3.

#### 3.2.3. 2,2′-((1,2-phenylenebis(azaneylylidene))bis(methaneylylidene))diphenol (HL_3_)

The ethanolic solution (25 mL) of salicylaldehyde (2.44 gm; 20 mmol), with an ethanolic solution of (25 mL) (1.08 gm; 10 mmol), was dissolved in methanol (5 mL) in the presence acetic acid as catalyst. The mixture was placed in a 100 mL round bottom flask connected with an external condenser and refluxed for 30 min. Bright yellow precipitates were obtained on cooling the reaction mixture, and the precipitates were filtered, washed with ethanol, and stored in vacuo over silica gel.

Bright yellow amorphous solid, yield, 82%; m.p. 161–163 °C. Elemental analysis of CHN for C_20_H_16_N_2_O_2_: C 75.93; H 5.10; N 8.86%. Found: C 75.82; H 4.95; N 8.71%. ^1^H-NMR spectrum (300 MHz, DMSO-*d*_6_) δ_H_, ppm: 6.92 (1H, m, Ph), 7.06 (1H, d, Ph, *J* = 8.1 Hz), 7.24 (1H, m, Ph), 7.35 (3H, m, Ph), 8.65 (1H, s, –CH=N–), 13.08 (1H, s, Phenolic); ^13^C-NMR spectrum (75 MHz, DMSO-*d*_6_) δ_C_, ppm: 117.58, 118.5 (C_2_, Ph), 119 (C_4_, Ph), 119.24 (C_9_, Ph), 119.73 (C_10_, Ph), 132.36 (C_3_, Ph), 133.4 (C_5_, Ph), 142.58 (C_8_, Ph), 161.36 (C_1_, –CH=N–), 163.73 (C_7_ Ph). Electrical Conductance (μS/cm) 1.06.

### 3.3. General Method for the Preparation of the Heteroleptic Metal Complexes (ML_1–3_Gly)

To well stirred ethanolic solutions (20 mL) of aldimine ligands (HL_1–3_), equimolar amounts of metal chlorides and phenyl glycine were gradually added, maintaining the reaction mixture temperature at 60–70 °C. The resulting precipitated products were filtered, washed, and dried in vacuum over silica gel.

CoL_1_Gly: Velvety brown amorphous solid, yield, 80%; m.p. 185–190 °C. Elemental analysis of CHN for C_28_H_22_CoN_3_O_4_: C 64.25; H 4.24; N 8.03%. Found: C 64.04; H 4.12; N 7. 89%. Electrical Conductance (μS/cm) 18.56.

NiL_1_Gly: Light green amorphous solid, yield, 78%; m.p. 312–316 °C. Elemental analysis of CHN for C_28_H_25_ClNiN_2_O_4_: C 61.41; H 4.60; N 5.12%. Found: C 60.87; H 4.55; N 5.02%. Electrical Conductance (μS/cm) 31.4.

CuL_1_Gly: Light blue amorphous solid, yield, 77%; m.p. >330 °C. Elemental analysis of CHN for C_28_H_25_ClCuN_2_O_4_: C 60.87; H 4.56; N 5.07%. Found: C 60.58; H 4.50; N 5.16%. Electrical Conductance (μS/cm) 13.42.

ZnL_1_Gly: White amorphous solid, yield, 81%; m.p. >350 °C. Elemental analysis of CHN for C_28_H_25_ClZnN_2_O_4_: C 60.67; H 4.55; N 5.05%. Found: C 60.63; H 4.32; N 5.00%. Electrical Conductance (μS/cm) 27.9.

CoL_2_Gly: Dark brown amorphous solid, yield, 65%; m.p. >250 °C. The elemental analysis of CHN for C_21_H_16_ClCoN_3_O_8_S: C 44.66; H 2.86; N 7.44%. Found: C 44. 47; H 2.76; N 7.40%. Electrical Conductance (μS/cm) 30.7.

NiL_2_Gly: Dark Green amorphous solid, yield, 74%; m.p. 333–338 °C. Elemental analysis of CHN for C_21_H_16_ClCoN_3_O_8_S: C 44.68; H 2.86; N 7.44%. Found: C 44.59; H 2.85; N 7.31%. Electrical Conductance (μS/cm) 4.69.

CuL_2_Gly: Brown amorphous solid, yield, 76%; m.p. 217–222 °C. Elemental analysis of CHN for C_21_H_16_ClCuN_3_O_8_S: C 44.30; H 2.83; N 7.38%. Found: C 44.28; H 2.76; N 7.21%. Electrical Conductance (μS/cm) 3.42.

ZnL_2_Gly: Light brown amorphous solid, yield, 80%; m.p. 280–282 °C. Elemental analysis of CHN for C_21_H_16_ClCuN_3_O_8_S: C 44.15; H 2.82; N 7.36%. Found: C 43.89; H 2.65; N 7. 31%. ^1^H-NMR spectrum (300 MHz, DMSO-*d*_6_) δ_H_, ppm: 3.50 (1H, s, SO_3_H), 3.34 (s, Me), 6.9 (1H, d, *J* = 8.7 Hz Ph), 7.1 (1H, t, *J* = 4.2 Hz, Ph), 7.3 (2H, d, *J* = 8.4 Hz, Ph), 7.6 (2H, d, *J* = 8.4 Hz, Ph), 8.7 (1H, s, C=N), 9.3 (1H, s, OH); ^13^C-NMR spectrum (75 MHz, DMSO-*d*_6_) δ_C_, ppm: 117.93 (C_12_), 119.37 (C_13_), 123.36 (C_4_, C_6_), 127.34 (C_3_, C_7_), 129.29 (C_2_), 131.16 (C-Cl, C_5_), 135.41 (C-NO_2_, C_10_), 136.60 (C_12_, C-SO_3_H), 139.25 (C_8_), 150.75 (C_9_, C- OH, Ph), 160.07 (C=N). Electrical Conductance (μS/cm) 27.9.

CoL_3_Gly: Velvety brown amorphous solid, yield, 79%; m.p. 185–190°C. Elemental analysis of CHN for C_28_H_22_CoN_3_O_4_: C 64.25; H 4.24; N 8.03%. Found: C 64.04; H 4.12; N 7.89%. Electrical Conductance (μS/cm) 31.7.

NiL_3_Gly: Maroon amorphous solid, yield, 78%; m.p. >346 °C. Elemental analysis of CHN for C_28_H_22_NiN_3_O_4_: C 64.28; H 4.24; N 8.03%. Found: C 63.75; H 3.99; N 8.01%. Electrical Conductance (μS/cm) 25.4.

CuL_3_Gly: Dark blue amorphous solid, yield, 90%; m.p. 225–235 °C. Elemental analysis of CHN for C_28_H_22_CuN_3_O_4_: C 63.69; H 4.20; N 7.83%. Found: C 63.58; H 4.01; N 7. 83%. Electrical Conductance (μS/cm) 13.5.

*ZnL_3_Gly:* Yellow amorphous solid, yield, 89%; m.p. 220–222°C. Elemental analysis of CHN for C_28_H_22_ZnN_3_O_4_: C 63.47; H 4.19; N 7.59%. Found: C 63.40; H 3.98; N 7. 93%. ^1^H-NMR spectrum (300 MHz, DMSO-*d*_6_) δ_H_, ppm: 6.5 (1H, m, Ph), 6.9 (1H, d, Ph, *J* = 8.1 Hz), 7.27 (1H, m, Ph), 7.4 (3H, m, Ph), 7.9 (1H, s, –CH=N–), 9.06 (1H, s, Phenolic); ^13^C-NMR spectrum (75 MHz, DMSO-*d*_6_) δ_C_, ppm: 113.42, 116.93 (C_2_, Ph), 119.89 (C_4_, Ph), 123.55 (C_9_, Ph), 119.73 (C_10_, Ph), 134.78 (C_3_, Ph), 136.69 (C_5_, Ph), 139.83 (C_8_, Ph), 163.29 (C_1_, –CH=N–), 172.73 (C_7_ Ph). Electrical Conductance (μS/cm) 3.57.

FTIR data of all the complexes and aldimine ligands are presented in Tables S4–S6 of the Supplementary Material.

### 3.4. The Stability Constants of Heteroleptic Complexes

Buffer solutions were prepared by using different volumes of 0.2 M sodium hydrogen phosphate prepared form 2.83g in (100 mL) in distilled water, and (0.1) M citric acid prepared by dissolving 1.92 gm in (100) mL distilled water [16]. 1 × 10^−3^ mol. The standard solutions ligands (HL_1_, HL_2_, HL_3_) and metal salt (CoCl_2_·6H_2_O, NiCl_2_·6H_2_O, CuCl_2_·2H_2_O, ZnCl_2_·2H_2_O) were prepared by dissolving the appropriate amounts in 100 mL of absolute ethanol. Metal stock solutions were further diluted with distilled water to prepare other standard solutions. 

### 3.5. Mechanistic Study

The reaction for the formation of the complex can be assumed as

(2) $$M_{m}L_{n}\to \mathrm{mM}+\mathrm{nL}$$

The equation for the dissociation of the complex, and stability constant (K_st_) can be expressed as:
M_m_L_n_→mM+nL
C
0
0Initial concentration(1−∝)C
m∝C
n∝CEquilibrium concentrations

(3) $$K_{\mathrm{st}}=\left(1-\propto \right)/m^{m}\times n^{n}\times {\left(\propto \right)}^{m+n}{\left(C\right)}^{m+n-1}$$

(4) $$K_{\mathrm{st}}=\left(1-\propto \right)C/{\left(m\propto C\right)}^{m}\times {\left(n\propto C\right)}^{n}=\left(1-\propto \right)C/m^{m}\times n^{n}\times {\left(\propto C\right)}^{m+n}$$

where ‘∝’ represents the degree of dissociation of the compound. ‘C’ is the initial concentration.

To find the stoichiometry and formation constant of the complex, metal ion solution and reagent solutions were mixed to give the following nine set of solutions:

Metal ion solution (mL) 0.5, 1.0, 1.5, 2.0, 2.5, 3.0, 3.5, 4.0 and 4.5

Reagent solution (mL) 4.5, 4.0, 3.5, 3.0, 2.5, 2.0, 1.5, 1.0 and 0.5.

The solutions were suitably diluted with distilled water up to 100 or 250 mL in volume to record the spectra. Job’s method was applied for stoichiometric ratio and stability constant determination of the complexes. The curve is obtained with a maximum corresponding to the specific value of mole fraction, which gives the stoichiometric mole fraction indicating the stoichiometry of the complex (M: L).

A_max_ is the absorbance value of the maxima at experimental curve represents the maximum quantity of the complex formed with a degree of dissociation (α), and A_ο_ is absorbance value corresponding to intersect point of theoretical straight lines. A_α_ is the absorbance value of the part of a dissociated concentration of the complex that represents the difference between A_ο_ and A_max_;

A_α_ = A_ο_ − A_max_ (5)

To estimate the stability constant, the degree of dissociation of complex (α) is calculated in the following manner

Aα = ε × b × (αC)(6)

α = Aα/ε × b × C(7)

where ε is molar absorptivity (calculated for each compound by using the reported method [31]), b is a cell thickness, and C is a concentration of the complex at stoichiometry point.

### 3.6. Antibacterial Studies

Studies on antibacterial activity of synthesized ligands and metal complexes were performed with slight changes in the reported protocol [22]. Six microbial strains viz. *B. cereus, S. aureus, K. oxytoca, E. coli, P. mirabilis*, and *P. aeruginosa* were used to study the antibacterial activity of ligands and their heteroleptic metal complexes. The microbes were grown in nutrient agar broth medium at 35 °C for 24 h using the agar-well diffusion method [13]. Muller Hinton’s agar plates were homogenized superficially by injecting 0.2 mL microbial cultures using pasteurized cotton plugs, followed by digging the wells into solidified agar with a sterilized cork borer (7 mm). Afterwards, 10 mg/mL solutions of test compounds dissolved in dimethylsulfoxide were shifted to the wells and left undisturbed for 30 min at 35°C for 24 h before incubation. The activities were assessed from the inhibition zones, whereas the bactericidal ciprofloxacin was the reference drug.

### 3.7. Antifungal Studies

The in vitro antifungal activities of all ligands and metal complexes were evaluated by employing the disc technique [22]. The antifungal screening against *A. niger*, *A. flevus* and *R. Stolonifer* were determined by potato dextrose agar (PDA) media prepared from washed-sliced skinned potatoes (250 g), dextrose (25 g), and agar (25 g) mixed in 1250 mL of distilled water. The pure fungal cultures were inoculated uniformly on the PDA solution in a Petri dish. Afterwards, 15 μg stock solutions were transferred to test samples (1 mg/mL) by liquefying 10 mg of the compound in 10 mL dimethylsulfoxide. These plates were inoculated followed by incubation for 48 h at 35 °C; then, the growth inhibition zone was observed as the sensitivities of fungal isolates with antibiogram. Diflucan (fluconazole) was the reference standard, while DMSO served as the negative control, and all the values < 7 mm were taken as inactive.

### 3.8. Solubilization Studies

#### 3.8.1. Solutions Preparation and Procedures for the Solubilization Studies

The solutions of the complexes were prepared in distilled water to make primary solutions, while secondary solutions were prepared by dissolving SLS in pre-micellar to a post-micellar concentration range (7–15 mM). 

#### 3.8.2. Electrical Conductivity (EC)

Electrical conductivity parameters were studied with the Hanna Cond. meter (HI-99301), ensuring the accuracy of up to ±0.5% and ∼±0.5 K. The electrode of conductivity meter was calibrated with KCl_(sol)_ to maintain the concentration in a particular range according to molar conductivity [32,33]. The differential conductivity was also measured.

The dissociation and free energy of micellization were calculated from conductivity data using Equations (8) and (9).

(8) $$\mathrm{Extent}\;\mathrm{of}\;\mathrm{dissociation}\;\left(\beta \right)=\frac{S_{2\;\left(\mathrm{Post}-\mathrm{micellar}\;\mathrm{slope}\right)}}{S_{1\;\left(\mathrm{Pre}-\mathrm{micellar}\;\mathrm{slope}\right)}}$$

(9) $$\mathrm{Free}\;\mathrm{Energy}\;\mathrm{of}\;\mathrm{Micellization}\;({\Delta G}_{m}^{{}^{\circ}})=\left(2-\beta \right)\mathrm{RT}\ln X_{\mathrm{CMC}}$$

In Equation (9), X_CMC_ = mole fraction of SLS at CMC, R = Universal gas constant (~8.314 Jmol^−1^·K^−1^), and T = absolute temperature. 

The results of the micellar parameters of complexes are summarized in Table 5.

#### 3.8.3. UV-Visible Spectroscopy

The interaction of metal complexes with SLS was estimated by measuring UV-visible absorbance of aqueous solutions of the metal complexes (1 mM). The extent and nature of the interaction between complexes and surfactant were revealed from the absorbance data at 298 K maintaining the accuracy control at ±0.5 K. In differential absorbance, the solution of the surfactant was taken as reference. These values of absorbance were then used for measuring partitioning and binding parameters using Equations (10)–(13).

The distribution of metal complexes in micellar and aqueous media was governed by Partition law according to Kawamura et al. 

The differential absorbance (∆A) was calculated using with relationship presented in Equation (10).

(10) $$\frac{1}{∆A}=\frac{1}{K_{C}∆A_{\infty }\left(C_{a}+C_{s}^{m_{{}^{\circ}}}\right)}+\frac{1}{∆A_{\infty }}\;$$

In Equation (10): K_c_ = partition constant; C_a_ = concentration of metal complexes; and ∆A_∞_ = differential absorbance on infinite dilution. In the expression $C_{s}^{m_{{}^{\circ}}}$ = C_s_ − CMC_o_; CMC_o_ = CMC of SLS in aqueous medium and C_s_ is SLS concentration. Partitioning coefficient K_x_ = K_c_n_w_ (n_w_ represent moles of water).

(11) $$\mathrm{Change}\;\mathrm{in}\;\mathrm{Free}\;\mathrm{energy}\;\mathrm{of}\;\mathrm{partition}\;(∆G_{p})=-\mathrm{RT}\ln K_{x}$$

The binding constant (K_b_) was computed with the help of Equation (12).

(12) $$\frac{C_{S}C_{a}}{∆A}=\frac{C_{S}}{∆\in l}+\frac{1}{K_{b}∆\varepsilon l}$$

In Equation (12), C_a_ is the concentration of the metal complex.

(13) $$\mathrm{Standard}\;\mathrm{binding}\;\mathrm{free}\;\mathrm{energy}\;\mathrm{change}\;(∆G_{b})=-\mathrm{RT}\ln K_{b}$$

The results of binding and partitioning parameters using the above equations are presented in Table 6

## 4. Conclusions

Heteroleptic 3D metal complexes having cobalt (II)/nickel (II)/copper (II)/zinc (II) with aldimine derivatives as a primary ligand and phenyl glycine as a secondary ligand were synthesized and characterized by different physico-chemical spectroscopic techniques. Elemental analysis and spectroscopic techniques allowed proposing the tetrahedral and octahedral geometry to the heteroleptic complexes synthesized. The spectroscopic analysis unveiled the tetrahedral geometry of the synthesized metal (II) complexes, except for ligand HL_3_ which exhibited octahedral geometry. The stability constants, antimicrobial and micellar solubilization studies were also conducted with synthesized heteroleptic complexes. The synthesized compounds generally showed antibacterial activity for all microbes, except Ni (II) complexes lacking sensitivity. The complexes of cobalt showed the highest stability as compared to other complexes. The antifungal activity of ligand (HL_1_, HL_2_, HL_3_), especially against *A.niger* and *R. stolonifer* fungal strains, was enhanced upon complexation to form heteroleptic cobalt complexes. All the complexes were screened for their solubilization studies in anionic micellar media, and only four complexes showed interaction with sodium lauryl sulphate (CoL_1–3_Gly and NiL_3_Gly). The CMC values obtained from the conductivity and UV-vis data were in good agreement.

## Figures and Tables

**Table 1:** Electronic spectral data of ligands (HL_1–3_) and heteroleptic complexes (ML_1–3_Gly).

Compound	Electronic Absorption Peaks (nm)	Type of Transition
HL_1_	347	n→π*, π→π*
CoL_1_Gly	724, 349.2, 262	d-d, CT
NiL_1_Gly	734, 348.2, 258	d-d, π→π*, n→π
CuL_1_Gly	734, 348.5, 260	d-d, π→π*
ZnL_1_Gly	349	Intra-ligand transition
HL_2_	441, 358	n→π*
CoL_2_Gly	690, 476, 456	d-d, CT (charge transfer)
NiL_2_Gly	675, 507, 406, 303, 262	d-d, CT, n→π*, π→π*
CuL_2_Gly	680, 532, 441, 426	d-d, CT
ZnL_2_Gly	445	Intra-ligand transition
HL_3_	334, 276	n→π*, π→π*
CoL_3_Gly	732, 419, 332, 256, 246	d-d, CT, n→π*, π→π*
NiL_3_Gly	640, 475, 377, 308, 246, 207	d-d, CT, n→π*, π→π*
CuL_3_Gly	657, 439	d-d, Intra-ligand, n→π*
ZnL_3_Gly	402, 293	Intra-ligand transition

**Table 2:** Stability Constants of heteroleptic complexes (ML_1–3_Gly).

Compound	K_st_ (kJ/mol)	Compound	K_st_ (kJ/mol)	Compound	K_st_ (kJ/mol)
CoL_1_Gly	5.47 × 10^12^	CoL_2_Gly	1.65 × 10^12^	CoL_3_Gly	3.34 × 10^13^
NiL_1_Gly	1.78 × 10^11^	NiL_2_Gly	1.75 × 10^12^	NiL_3_Gly	4.58 × 10^12^
CuL_1_Gly	1.9 × 10^9^	CuL_2_Gly	7.5 × 10^7^	CuL_3_Gly	6.25 × 10^5^
ZnL_1_Gly	2.95 × 10^10^	ZnL_2_Gly	8.21 × 10^8^	ZnL_3_Gly	1.22 × 10^5^

**Table 3:** Antibacterial data of HL_1–3_, Gly and their heteroleptic complexes at 1 mg/mL concentration.

Compound	Minimum Zone of Inhibition (mm)					
	*Bacillus cereus*	*Escherichia coli*	*Klebsiella oxytoca*	*Pseudomonas aeruginosa*	*Staphylococcus aureus*	*Proteus mirabilis*
HL_1_	24.5	17.3	27.3	17.3	13.9	16.8
HL_2_	22.3	16.2	24.9	16.5	14.6	19.4
HL_3_	24.1	15.5	23.6	18.4	16.1	20.3
Gly	14.9	13.2	23.0	11.5	13.7	17.0
CoL_1_Gly	31.0	19.5	24.4	18.6	20.1	26.0
NiL_1_Gly	29.0	21.0	R	R	23.0	18.5
CuL_1_Gly	21.0	28.5	21.5	23.1	23.0	27.3
ZnL_1_Gly	27.7	23.0	23. 6	30.3	14.1	18.3
CoL_2_Gly	28.0	18.0	21.9	17.3	19.1	21.0
NiL_2_Gly	26.9	20.3	R	R	21.5	27.6
CuL_2_Gly	19.2	24.1	22.9	24.0	23.0	26.0
ZnL_2_Gly	26.1	22.4	22.2	28.8	17.1	16.0
CoL_3_Gly	32.2	21.6	24.4	18.6	20.1	28.1
NiL_3_Gly	30.3	23.6	R	R	25.4	30.0
CuL_3_Gly	23.1	29.7	23.1	25.0	26.2	29.2
ZnL_3_Gly	27.4	24.4	25.1	32.1	16.4	18.3
^+^ Ciprofloxacin	33.0	32.0	36.0	26.0	29.0	23.0
–(CH_3_)_2_SO	R	R	R	R	R	R

R: No bacterial growth inhibition was observed; ^+^: standard drug.

**Table 4:** Antifungal data of HL, Gly and Gly’s heteroleptic complexes at 1 mg/mL concentration.

Compound	Minimum Zone of Inhibition (mm)		
	*A. niger*	*A. flevus*	*R. stolonifer*
HL_1_	22.7	28.7	26.3
HL_2_	23.2	29.1	29.4
HL_3_	24.7	27.6	28.6
Gly	16.0	19.8	13.6
CoL_1_Gly	25.6	24.1	26.5
NiL_1_Gly	14.5	24.2	–
CuL_1_Gly	22.2	24.1	24.7
ZnL_1_Gly	17.3	25.3	–
CoL_2_Gly	24.6	29.3	27.9
NiL_2_Gly	14.4	23.1	–
CuL_2_Gly	24.2	24.5	26.1
ZnL_2_Gly	13.0	26.0	–
CoL_3_Gly	25.1	27.1	28.6
NiL_3_Gly	16.5	24.1	–
CuL_3_Gly	23.9	27.5	25.5
ZnL_3_Gly	18.0	28.1	–
^+^ Fluconazole	36.0	29.0	39.3
–(CH_3_)_2_SO	–	–	–

^+^: standard drug.

**Table 5:** Calculated micellar parameter for the interaction of complexes with SLS.

System	CMC (mM)	∆G_m_ (kJ/mol)	β
SLS	8.3	−38.39	0.243
CoL_1_Gly/SLS	10.52	−33.79	0.426
CoL_2_Gly/SLS	9	−28.11	0.7
CoL_3_Gly/SLS	9.56	−27.11	0.737
NiL_3_Gly/SLS	10.83	−31.072	0.532

**Table 6:** Partitioning and binding parameters of selected complexes in micellar media.

Complex	K_x_ (dm^3^/mol)	∆G_p_ (kJ/mol)	K_b_ (dm^3^/mol)	∆G_b_ (kJ/mol)
CoL_1_Gly	137	−12.19	190	−13
CoL_2_Gly	27,400	−25.31	325	−14.25
CoL_3_Gly	50,000	−26.8	58.5	−10.08
NiL_3_Gly	956	−17	80	−10.85

## Supplementary Materials

The detailed physico-chemical analysis of the ligands and complexes is reported in Tables S1–S3. The FTIR analyses details are given in Tables S4–S6. Selected NMR and FTIR spectra of ligands and complexes are reported in Figures S1–S16, while selected electronic spectra of ligands and complexes are given in Figure S17.
