# Supplementary material for: New Heteroleptic 3D Metal Complexes: Synthesis, Antimicrobial and Solubilization Parameters
Source: Molecules. 2020 Sep 16;25(18):4252. doi: 10.3390/molecules25184252 (PMC7570477; doi:10.3390/molecules25184252)
Supplement: Supplementary file 1 [file molecules-25-04252-s001.pdf]

Supplementary Material

New Heteroleptic 3D Metal Complexes: Synthesis, Antimicrobial and Solubilization Parameters

Muhammad Babar Taj [1,2,*,†], Muneera D. F. Alkahtani [3,*], Uzma Ali [4,†], Ahmad Raheel [4], Walla Alelwani [5], Afnan M. Alnajeebi [5], Nouf Abubakr Babteen [5], Sadia Noor [6,*] and Heba Alshater [7]

1 Department of Chemistry, Islamia University of Bahawalpur, Bahawalpur 63100, Pakistan
2 Department of Chemistry, University of Sahiwal, Sahiwal 57000, Pakistan
3 Department of Biology, College of Science, Princess Nourah Bint Abdulrahman University, Riyadh 11675, Saudi Arabia
4 Department of Chemistry, Quaid-e-Azam University, Islamabad 44000, Pakistan; uzmaali@hotmail.com (U.A.); ahmadraheel001@gmail.com (A.R.)
5 Department of Biochemistry, Faculty of Science, University of Jeddah, Jeddah 80203, Saudi Arabia; welwani@uj.edu.sa (W.A.); Amalnajeebi@uj.edu.sa (A.M.A.); Nababteen@uj.edu.sa (N.A.B.)
6 Department of Chemistry, University of Agriculture, Faisalabad 38040, Pakistan
7 Department of Forensic Medicine and Clinical Toxicology, Menoufia University, Shbien El-Kom 32511, Egypt; hebaalshater@yahoo.com
* Correspondence: dr.taj@iub.edu.pk or drbabartaj@gmail.com (M.B.T.); mdf.alkahtani@gmail.com (M.D.F.A.); sadiaa613@gmail.com (S.N.); Tel.: +92-300-7542669 (M.B.T.); +92-333-6531365 (S.N.)
† These authors contributed equally to this work.

Table 1. Physical and Analytical Data of HL_1 and its heteroleptic complexes (ML_1Gly).

Compound	Colour	Melting Temp (°C)	Elemental analysis (%) found (calc.)				Electrical Conductance µS/cm
			C	H	N	Metal	
HL_1 $C_{20}H_{18}ClNO_2$	Yellow	250-254	70.65 (70.69)	5.28 (5.34)	3.90 (4.12)		3.55
CoL_1Gly $C_{28}H_{25}ClCoN_2O_4$	Pink	>350	60.78 (61.38)	4.49 (4.60)	5.31 (5.11)	10.57 (10.76)	18.56
NiL_1Gly $C_{28}H_{25}ClNiN_2O_4$	Light green	312-316	60.87 (61.41)	4.55 (4.60)	5.02 (5.12)	10.61 (10.72)	31.4
CuL_1Gly $C_{28}H_{25}ClCuN_2O_4$	Light Blue	>330	60.58 (60.87)	4.50 (4.56)	5.16 (5.07)	11.41 (11.50)	13.42
ZnL_1Gly $C_{28}H_{25}ClZnN_2O_4$	White	>350	60.63 (60.67)	4.32 (4.55)	5.00 (5.05)	11.66 (11.79)	27.9

Table 2. Physical and Analytical Data of HL_2 and its heteroleptic complexes (ML_2Gly).

Compound	Colour	Melting Temp (°C)	Elemental analysis (%) found (calc.)				Electrical Conductance µS/cm
			C	H	N	Metal	
HL_2 $C_{13}H_9ClN_2O_6S$	Brown	125-128	43.55 (43.77)	2.50 (2.54)	7.67 (7.85)		-
CoL_2Gly $C_{21}H_{16}ClCoN_3O_8S$	Dark brown	> 250	44. 47 (44.66)	2.76 (2.86)	7.40 (7.44)	10.39 (10.43)	30.7
NiL_2Gly $C_{21}H_{16}ClNiN_3O_8S$	Dark Green	333-338	44. 59 (44.68)	2.85 (2.86)	7.31 (7.44)	10.22 (10.40)	4.69
CuL_2Gly $C_{21}H_{16}ClCuN_3O_8S$	Brown	217-222	44.28 (44.30)	2.76 (2.83)	7.21 (7.38)	11.01 (11.16)	3.42

ZnL$_2$Gly $C_{21}H_{16}ClCuN_3O_8S$	Light brown	280-282	43.89 (44.15)	2.65 (2.82)	7.31 (7.36)	11.34 (11.44)	27.9

Table 3. Physical and Analytical Data of HL$_3$ and its heteroleptic complexes (ML$_3$Gly).

Compound	Colour	Melting Temp (°C)	Elemental analysis (%) found (calc.)				Electrical Conductance µS/cm
			C	H	N	Metal	
HL$_3$ $C_{20}H_{16}N_2O_2$	Bright Yellow	161-163	75.82 (75.93)	4.95 (5.10)	8.71 (8.86)		1.06
CoL$_3$Gly $C_{28}H_{22}CoN_3O_4$	Velvety brown	185-190	64.04 (64.25)	4.12 (4.24)	7.89 (8.03)	11.11 (11.26)	31.7
NiL$_3$Gly $C_{28}H_{22}NiN_3O_4$	Maroon	> 346	63.75 (64.28)	3.99 (4.24)	8.01 (8.03)	11.01 (11.22)	25.4
CuL$_3$Gly $C_{28}H_{22}CuN_3O_4$	Dark blue	225-235	63.58 (63.69)	4.01 (4.20)	7.83 (7.96)	11.99 (12.03)	13.5
ZnL$_3$Gly $C_{28}H_{22}ZnN_3O_4$	Yellow	220-222	63.40 (63.47)	3.98 (4.19)	7.59 (7.93)	12.03 (12.34)	3.57

Table 4. FTIR Data (cm^{-1}) of HL$_1$ and its heteroleptic complexes (ML$_1$Gly).

Type of Vibration (cm^{-1})	Water of hydration	ν(N-H) Asym. /Sym.	ν(C=O)	ν(C=N) ~shift	ν(C-O)	ν(COO$^-$) asym.	ν(COO$^-$) sym.	ν-(M-O)	ν-(M-N)
Ligand HL$_1$	-	-	1647 (s)	1591 (s)	1215 (m)	-	-	-	-
Co(II) L$_1$- PhGly (ternary)	3360 (w)	3246 (w) 2976 (b, w)	1645 (m)	1585 (s)	1201 (m)	1556 (s)	1357 (s)	- 615 (s)	597 (m) 549 (m)
Ni(II) L$_1$- PhGly (ternary)	3363 (w)	3224 (w) 3149 (w)	1643 (w)	1581 (s)	1199 (m)	-	1409 (s)	646 (s) 617 (s)	586 (s) 509 (s)
Cu(II) L$_1$- PhGly (ternary)	3334 (w)	3217 (w) 3047 (w)	1622 (s)	1562 (s)	-	-	1365 (s)	609 (s)	542 (m) 459 (m)
Zn(II) L$_1$- PhGly (ternary)	3315 (w)	3255 (w) 3034 (w)	1618 (s)	1560 (s)	1197 (m)	-	1377 (s)	648 (m) 615 (s)	555 (m) 478 (s)

Table 5. FTIR Data (cm^{-1}) of HL$_2$ and its heteroleptic complexes (ML$_2$Gly).

Type of Vibration (cm^{-1})	Water of hydration	ν(=C-H) aromatic	ν(C=N) ~shift	ν-(NO$_2$)	ν-(Sulfonic acid)	ν(C-H) sp^2 Aromatic (o.o.p bending)	ν-(S-O)	νCOO$^-$ asym. /sym.	ν-(M-O)	ν-(M-N)
Ligand HL$_2$	3379 (w)	3027 (w)	1627 (s)	1370 (s) 1568 (s)	1205 (m) 1276 (s)	910 (m) 806 (s)	658 (m)	-	-	-

Co(II) L_2-PhGly (ternary)	3358 (w)	3062 (w)	1651 (s)	1556 (s) 1404 (s)	1192 1286 (s)	931 (m) 754 (s) 698(s)	667 (m)	1585 (s) 1361 (s)	657 (m) 609 (s)	555 (m) 486 (s)
Ni(II)L_2-PhGly (ternary)	3363 (w)	3062 (w)	1595 (s)	1475 (s) 1404 (s)	1192 (m) 1118 (s)	921 (m) 817 (s) 696 (s)	665 (s)	1581 1294 (s)	644 (s) 584 (s)	484 (m) 451 (s)
Cu(II)L_2-PhGly (ternary)	3332 (w)	3039 (w)	1622 (s)	1493 (m) 1402 (m)	1139 (s) 1105	921 (m) 752(s) 696 (s)	671 (m)	1562 (s) 1357 (s)	615 (s) 597 (m)	- 441 (s)
Zn(II)L_2-PhGly (ternary)	3313 (w)	3034 (w)	1616 (s)	1485 (s) 1409 (s)	1197(m) 1292 (s)	927 (m) 746 (s) 696 (s)	646 (m)	1562 (s) 1377 (s)	624 (s) 590 (m)	542 (m) 468 (s)

Table 6. FTIR Data (cm^{-1}) of HL_3 and its heteroleptic complexes (ML_3Gly).

Type of Vibration (cm^{-1})	Water of hydration	ν(=C-H) aromatic	ν(CH) sp^3	ν(C=N) ~shift	ν-(C-O)	ν(C-H) sp^2 aromatic (o.o.p bending)	ν(COO^-) asym.	ν(COO^-) sym.	ν-(M-O)	ν-(M-N)
Ligand HL_3	O-H phenolic 3430 (w)	3055 (w)	-	1608 (s)	1274 (s)	754 (s) 854 (m)	-	-	-	-
Co(II)L_3-PhGly (ternary)	3414	-	-	1630 (s)	1200 (s)	751 (s) 855 (m)	1521(s)	1357 (s)	597 (s) 511 (m)	475 (s) 412 (s)
Ni(II)L_3-PhGly (ternary)	3388 (b, w)	-	2896 (w)	1601 (s)	1233 (m)	752 (s) 847 (m)	1517 (s)	1370 (s)	543 (s) 507 (m)	457 (s) 409 (s)
Cu(II)L_3-PhGly (ternary)	3406 (b, w)	2906 (w)	2887 (w)	1576 (s)	1200 (m)	694 (s) 728 (s) 854 (m)	1519 (s)	1356 (s)	595 508 (s)	472 (m)
Zn(II)L_3-PhGly (ternary)	-	-	2895 (w)	1613 (s)	1244 (m)	747 (s) 854 (m)	1528 (s)	1323 (s)	597 510 (s)	490 437 (m)

Comparison of Zn(II) complexes.

Type of Vibration (cm^{-1})	Water of hydration	ν($-NH_2$) sym./ asym.	ν(CH) sp^3 and ν(=C-H) Phe.	ν(C=N) ~shift	ν-(C-O)	ν(C-H) sp^2 aromatic (o.o.p bending)	ν(COO^-) asym.	ν(COO^-) sym.	ν-(M-O)	ν-(M-N)
Ph-Gly	-	2975, 3167 (w)	2880 (w)	-	-	694 (s) 728 (s)	1576 (s)	1355 (s)	-	-
Zn(II)-Ph-Gly (binary)	3313 (w)	3062 3256 (w)	3034 (w)	-	-	697 (s) 746 (s)	1560 (s)	1376 (s)	586 (s)	460 (s)
Ligand HL_3	O-H phenolic 3430 (w)	-	-	1608 (s)	1274 (s)	754 (s) 854 (m)	-	-	-	-

ZnL_3 (binary)	3453 (w)	-	3007 (w)	1612 (s)	1244 (m)	746 (s) 854 (m)	-	-	540 (s)	488 (s)
ZnL_3- PhGly (ternary)	-	2965 (w) masked	2895 (w)	1613 (s)	1244 (m)	747 (s) 854 (m)	1528 (s)	1323 (s)	597 510 (s)	490 437 (m)

Selected NMR and FTIR spectra's of synthesized ligands and mixed complexes

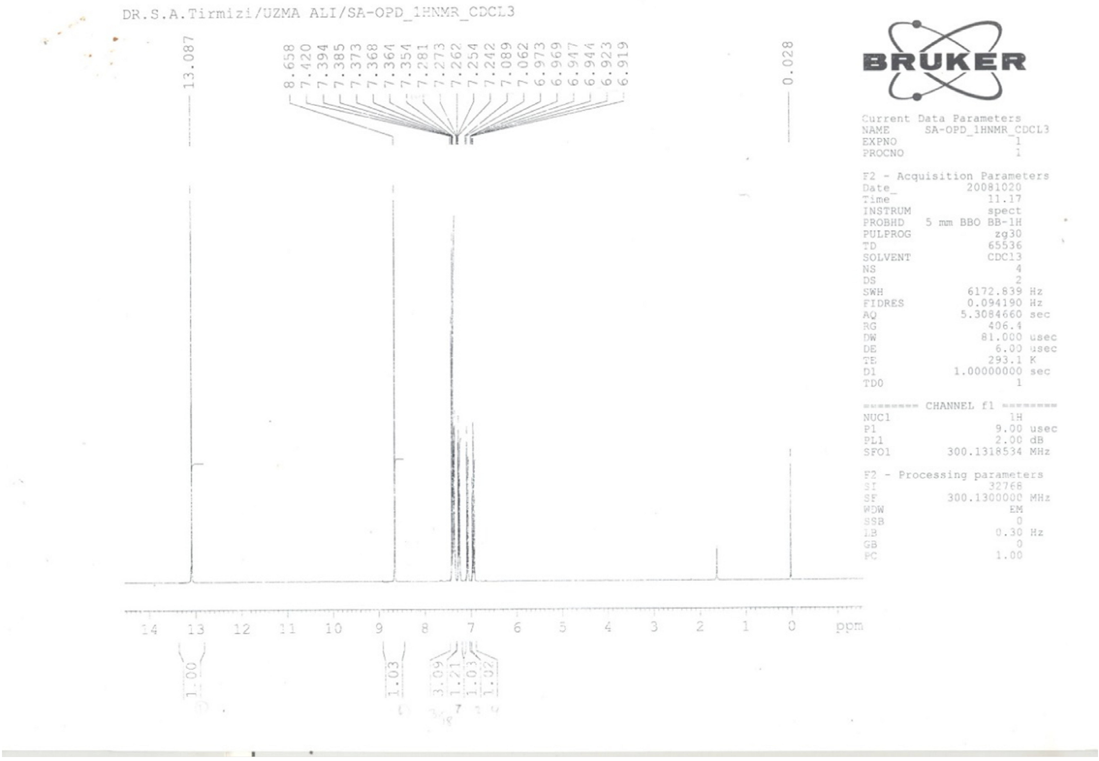

Figure 1. ^{1}H NMR spectrum of HL_3.

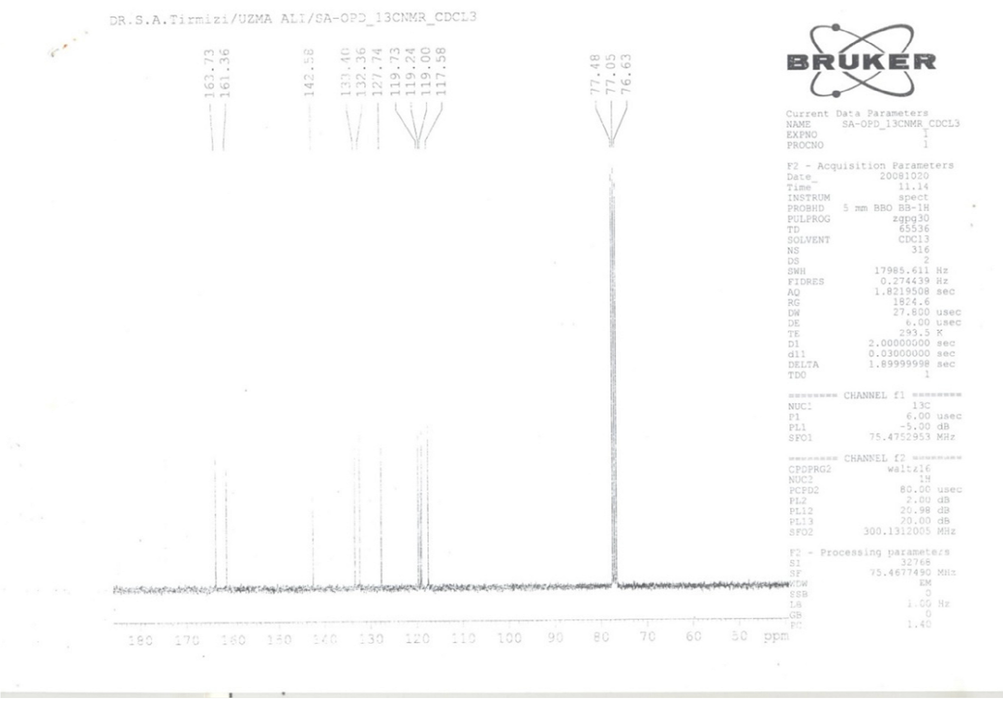

Figure 2. ^{13}C NMR spectrum of HL_3.

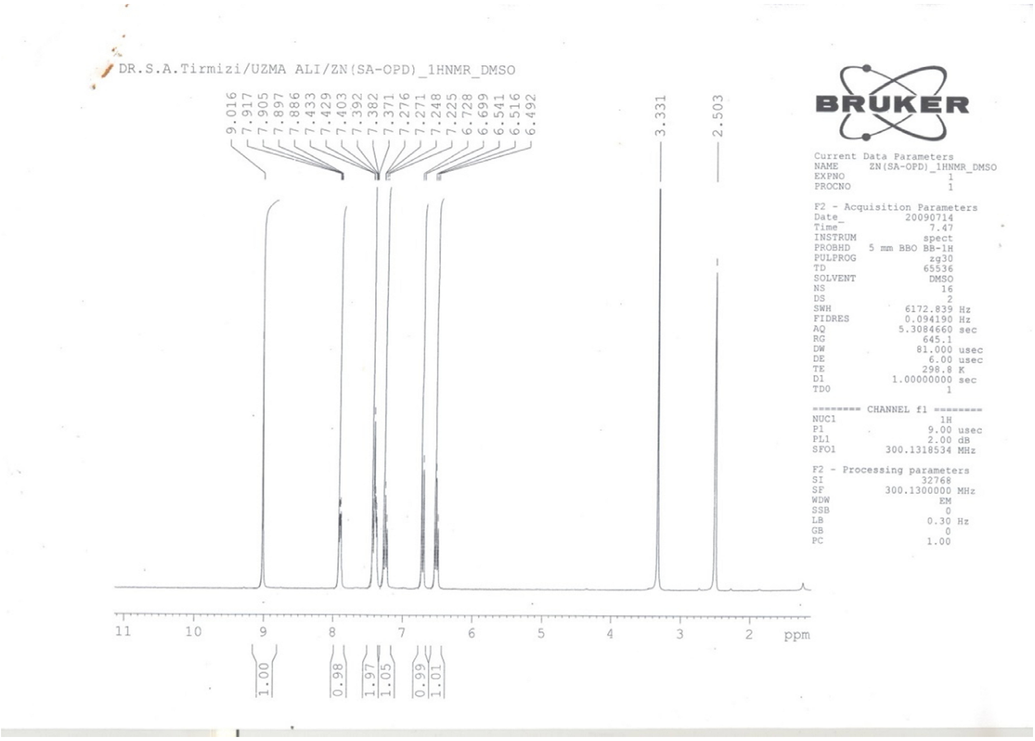

Figure 3. ^{1}H NMR spectrum of ZnL_3Gly.

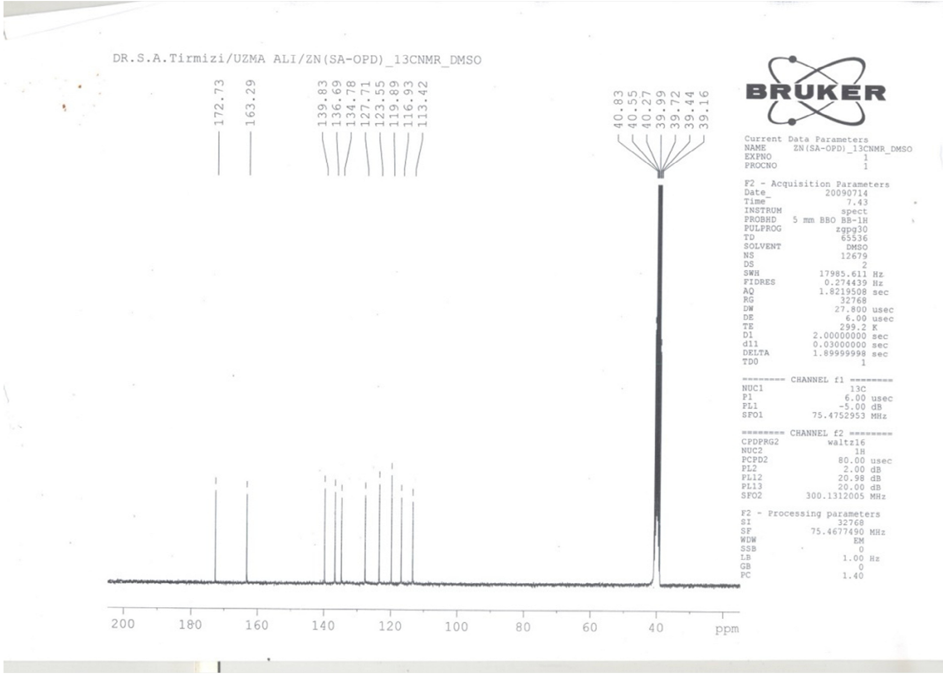

Figure 4. ^{13}C NMR spectrum of ZnL_3Gly.

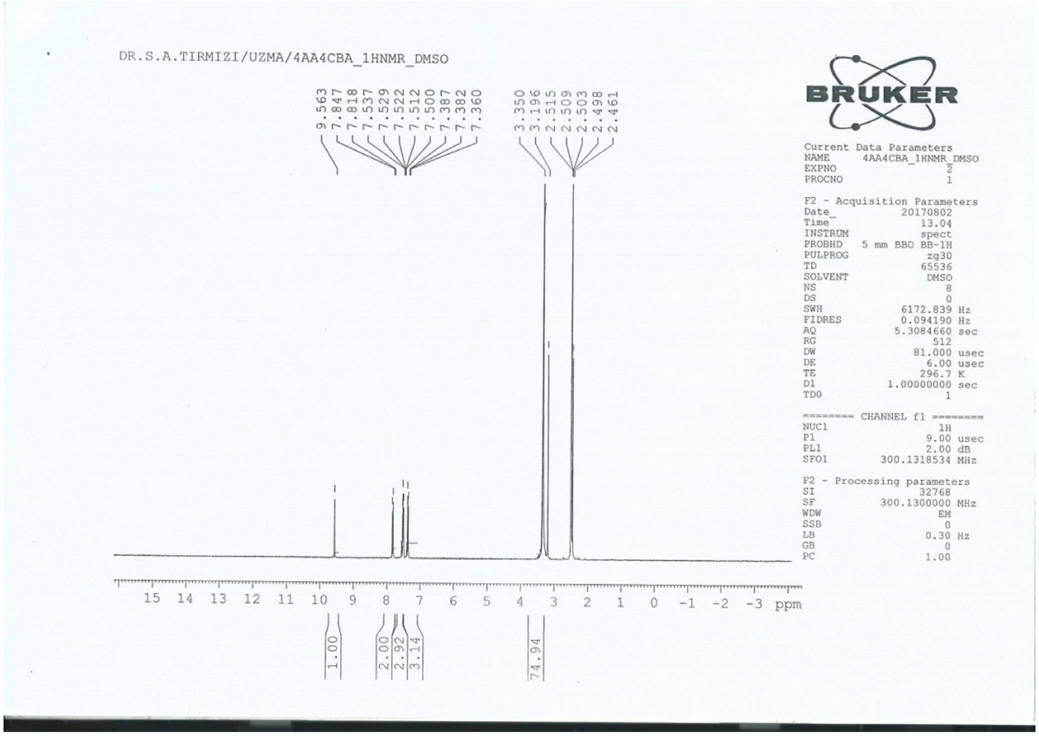

Figure 5. ^{1}H NMR spectrum of HL_1.

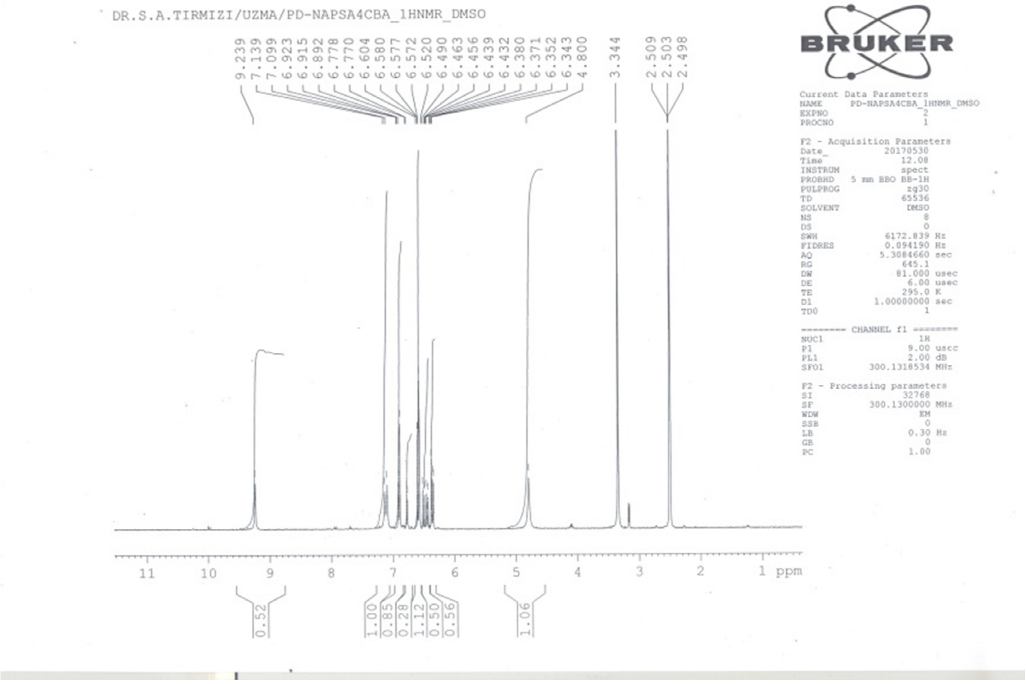

Figure 6. ^{1}H NMR spectrum of HL_2.

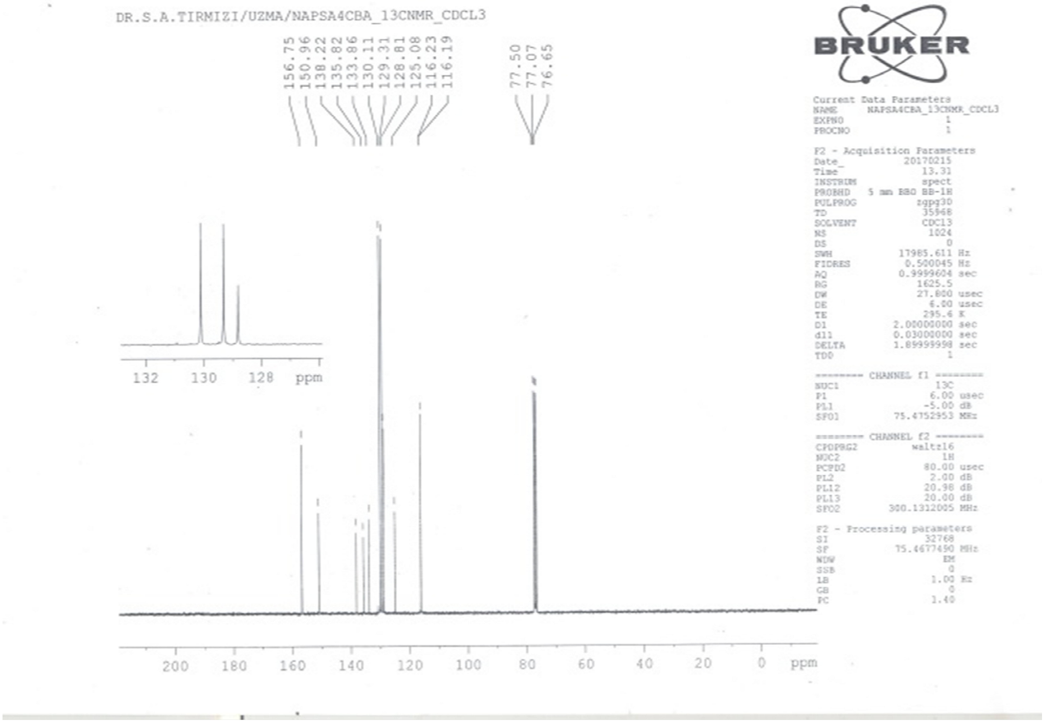

Figure 7. ^{13}C NMR spectrum of HL2.

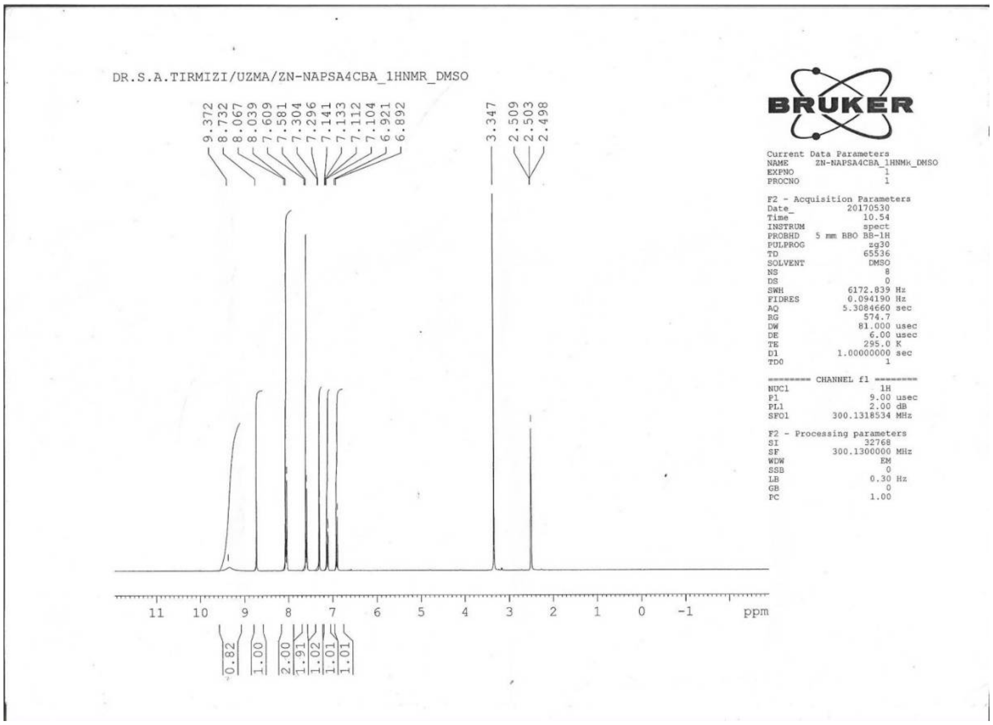

Figure 8. ^{1}H NMR spectrum of ZnL_2Gly.

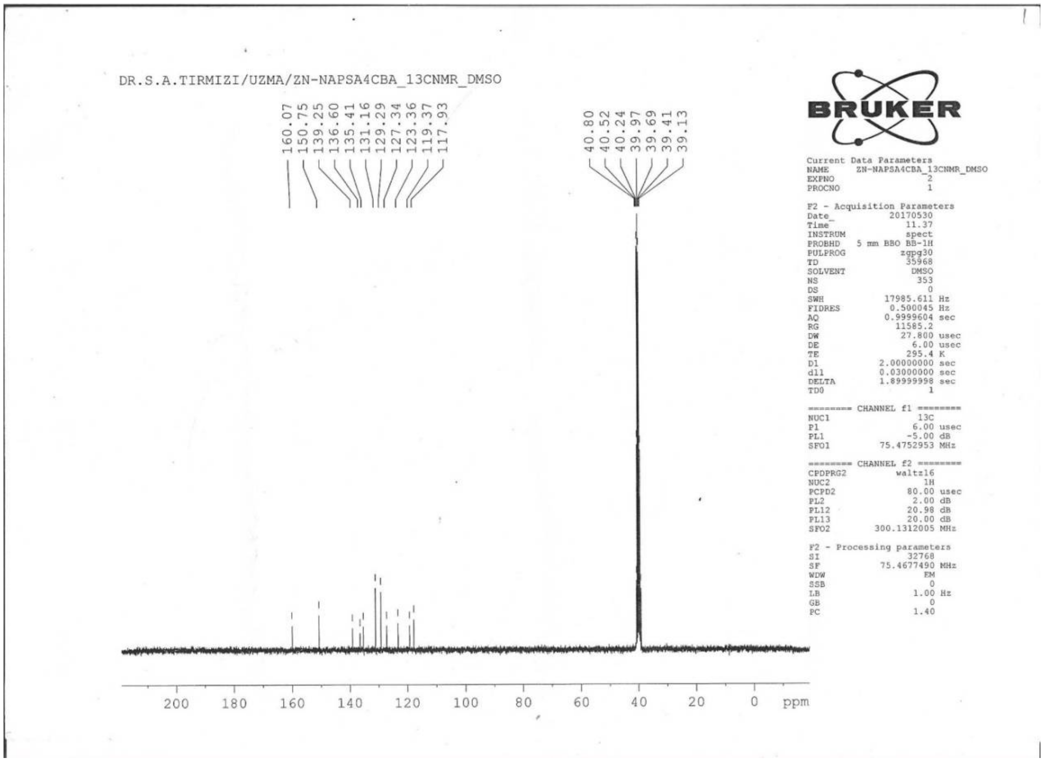

Figure 9. ^{13}C NMR spectrum of ZnL_2Gly.

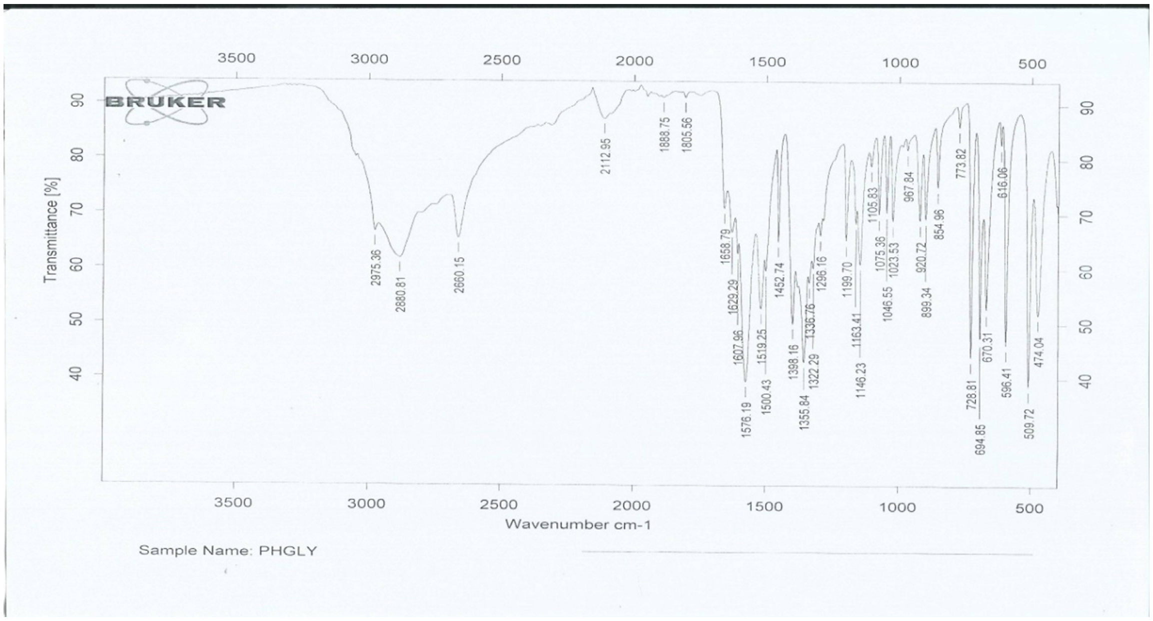

Figure 10. FTIR spectrum of α-phenyl glycine.

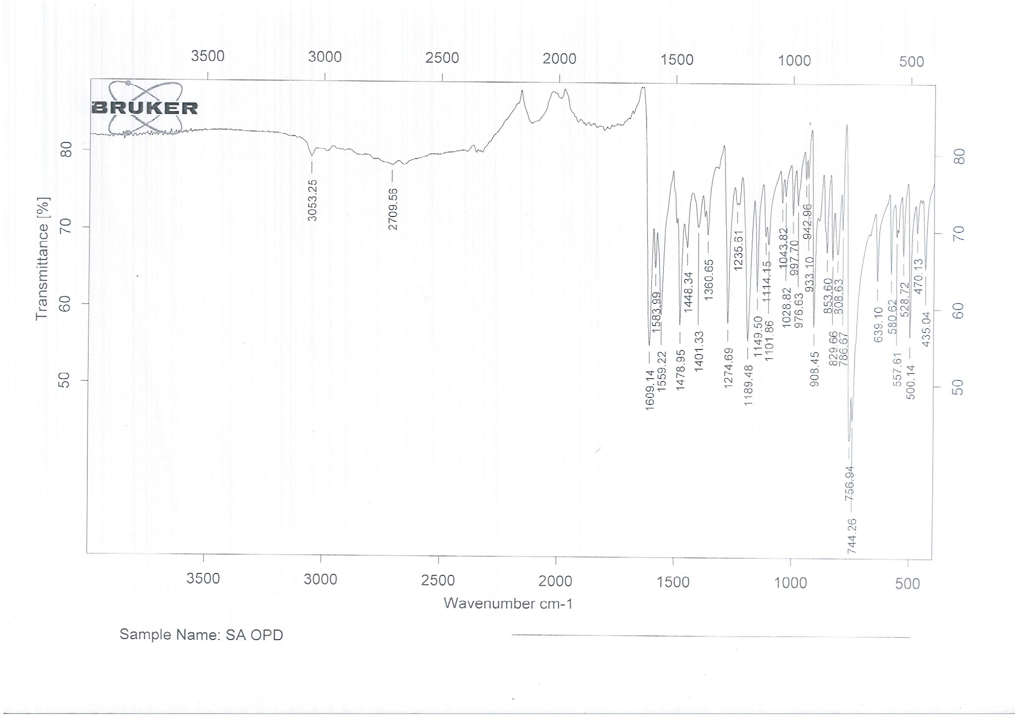

Figure 11. FTIR spectrum of HL_3.

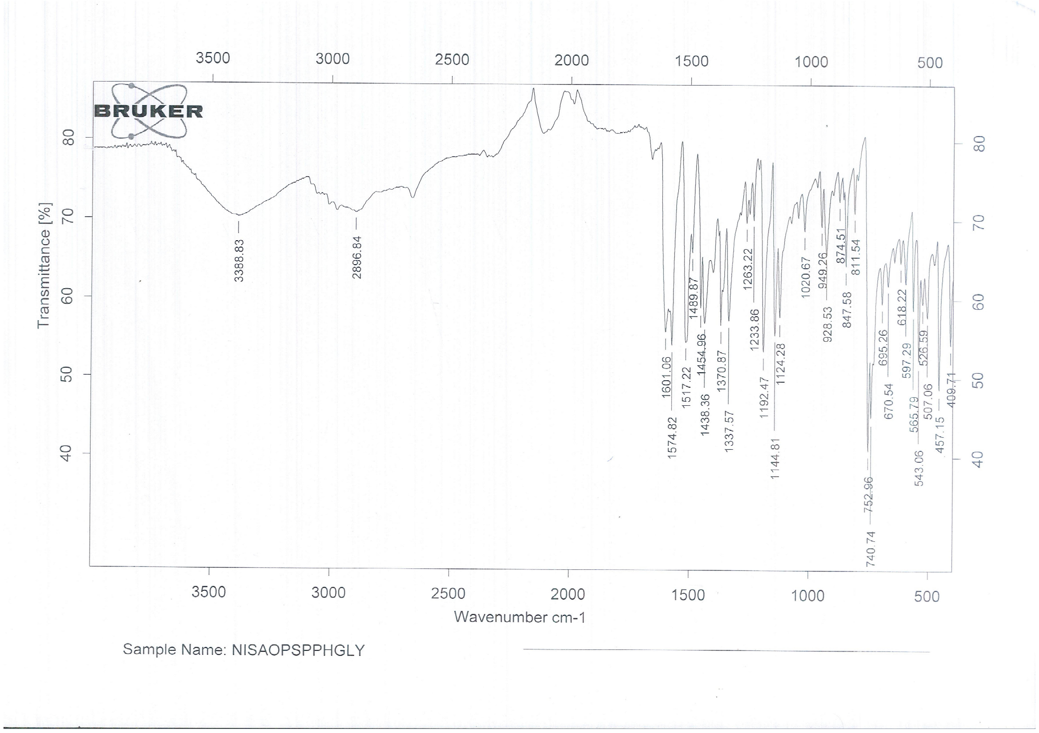

Figure 12. FTIR spectrum of NiL_3Gly.

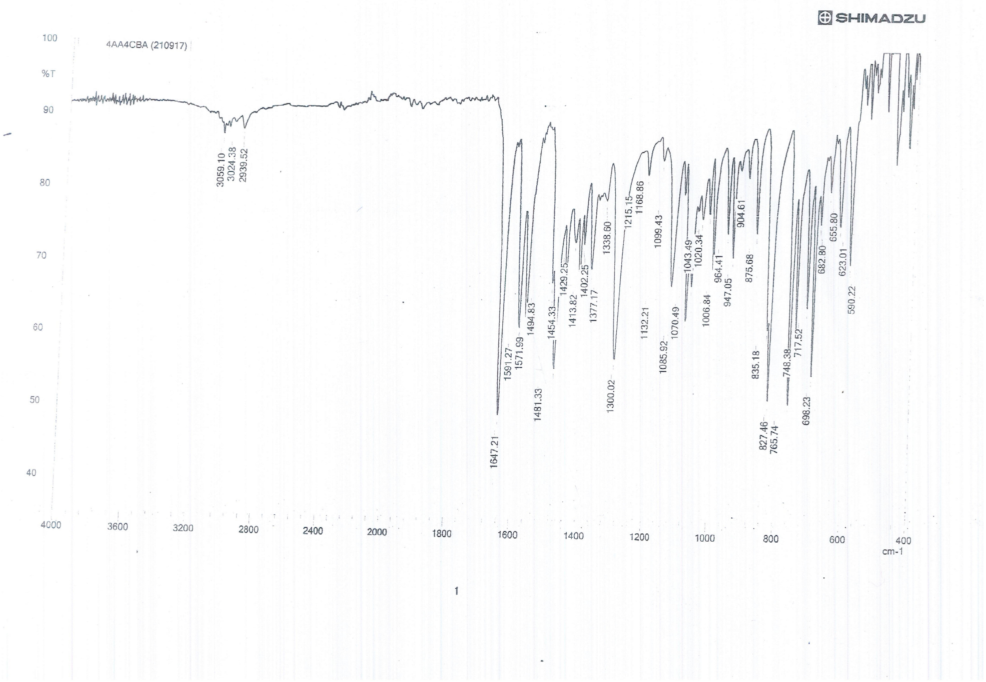

Figure 13. FTIR spectrum of HL_1.

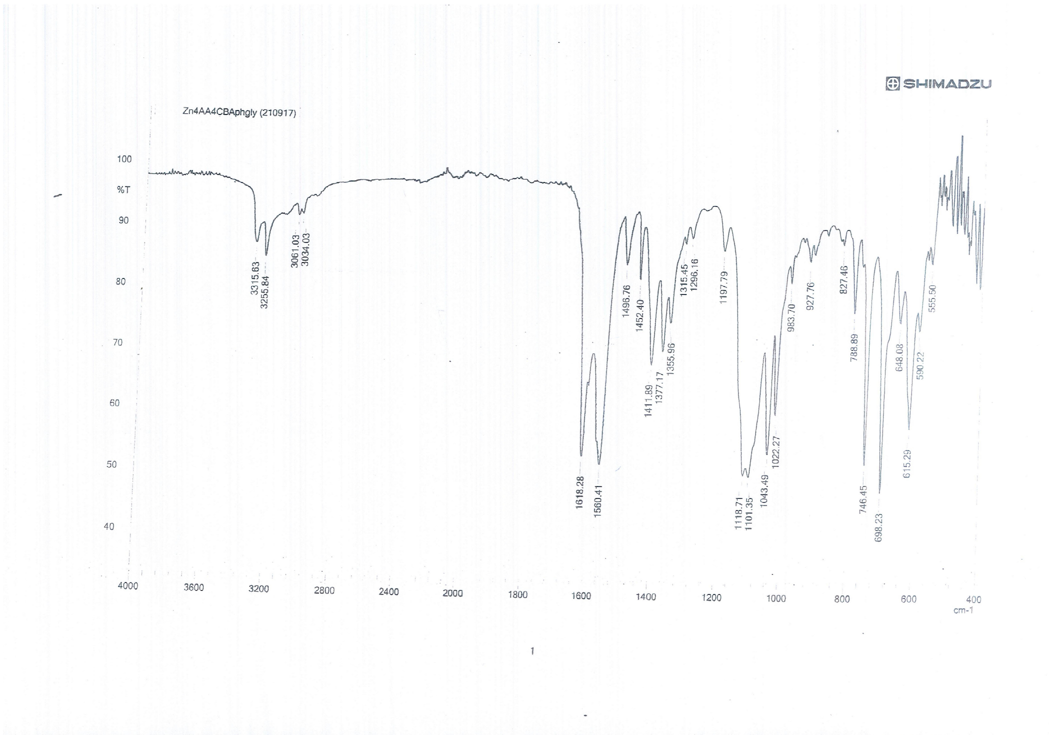

Figure 14. FTIR spectrum of ZnL_1Gly.

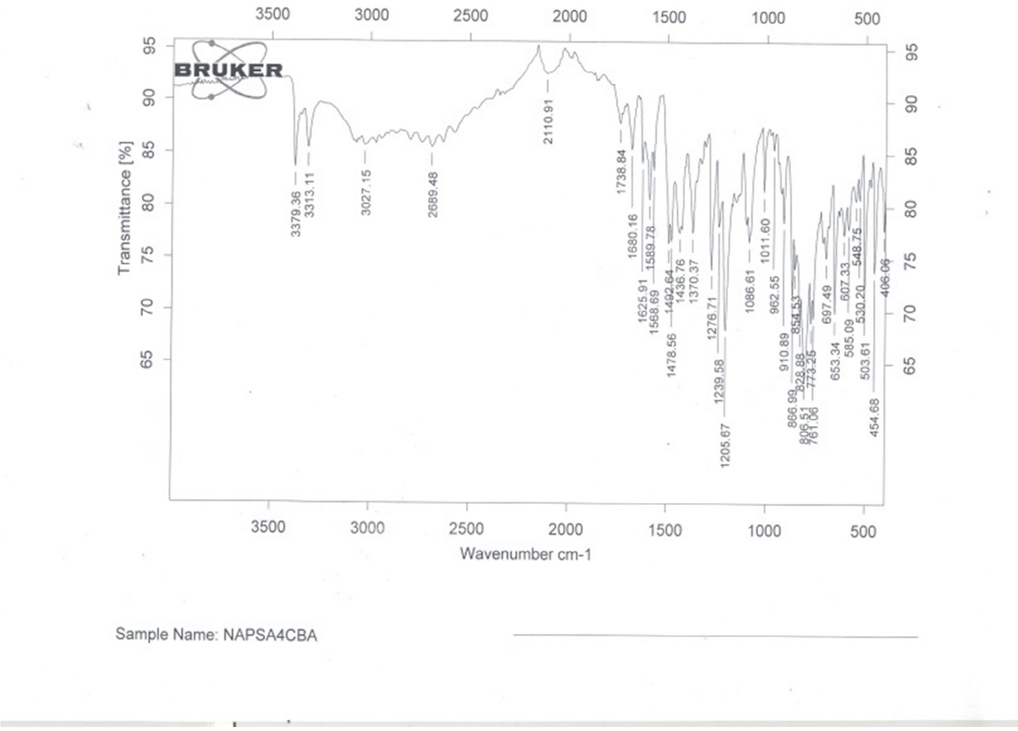

Figure 15. FTIR spectrum of HL_2.

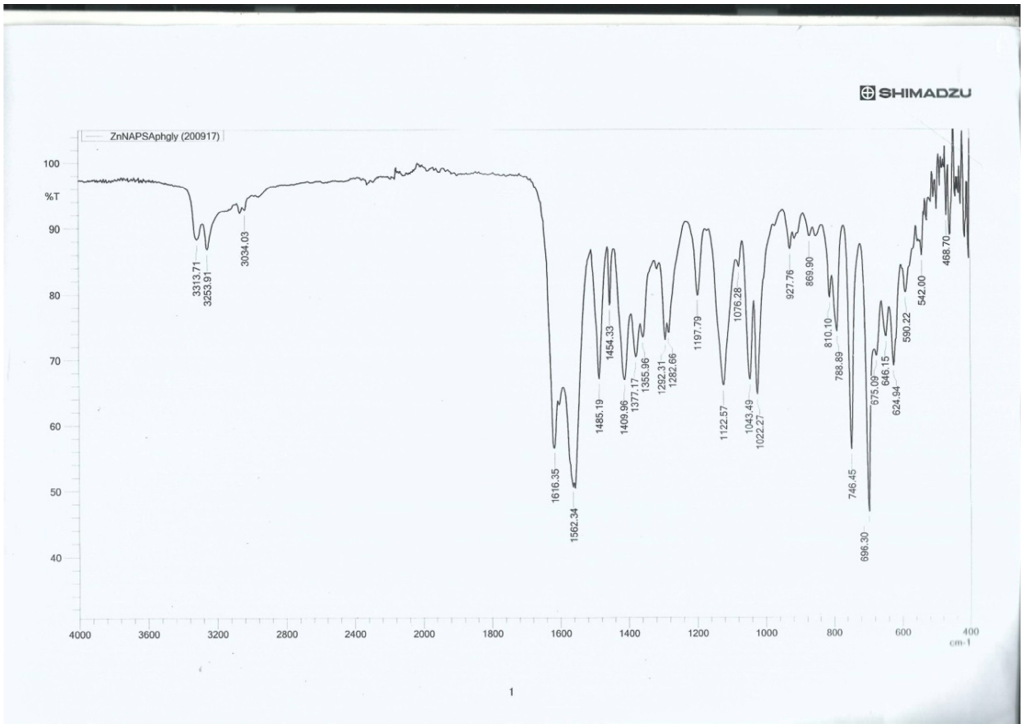

Figure 16. FTIR spectrum of ZnL_2Gly.

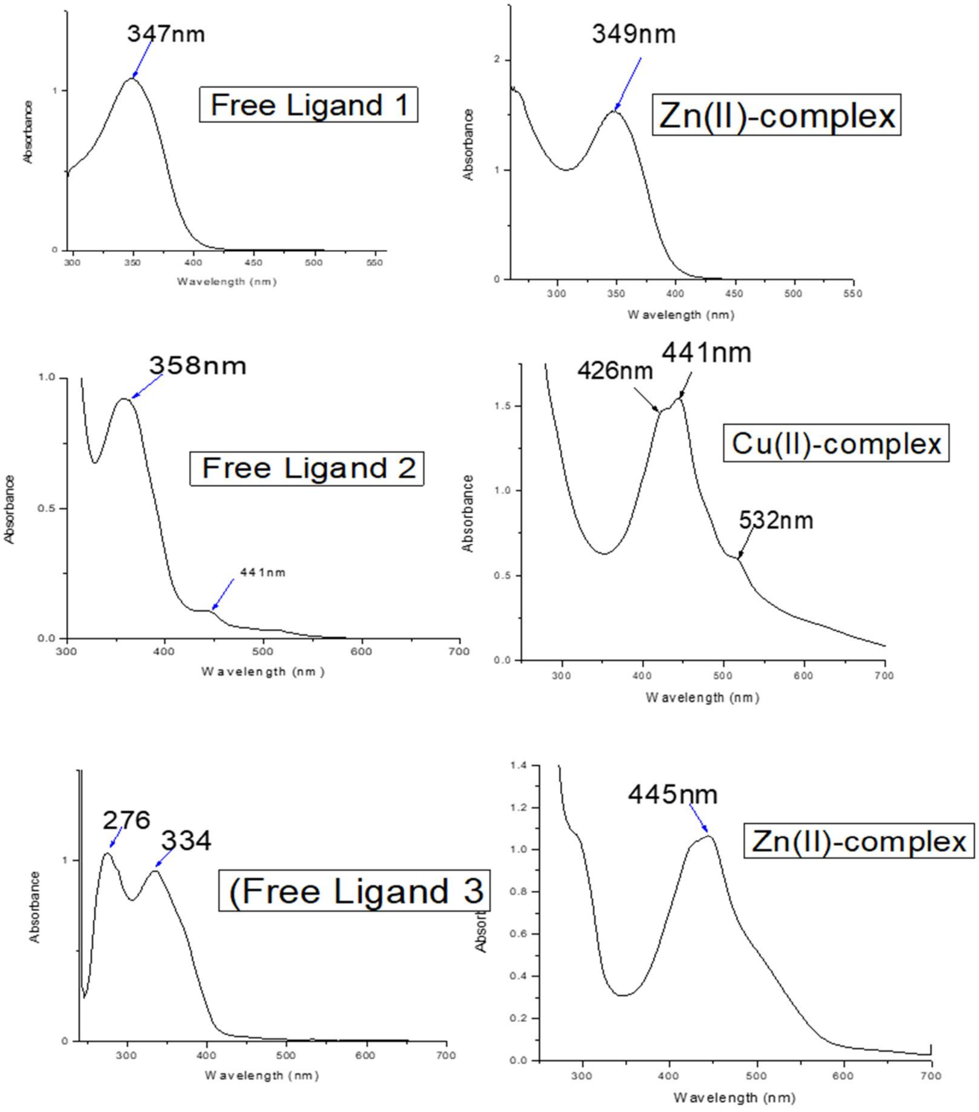

Figure 17. Electronic spectra of ligands (HL_1, HL_2, HL_3) and selected spectra of metal complexes.
